# Muscle and Serum Metabolomics for Different Chicken Breeds under Commercial Conditions by GC–MS

**DOI:** 10.3390/foods10092174

**Published:** 2021-09-14

**Authors:** Chengkeng Tan, Jinap Selamat, Nuzul Noorahya Jambari, Rashidah Sukor, Suganya Murugesu, Alfi Khatib

**Affiliations:** 1 Food Safety and Food Integrity (FOSFI), Institute of Tropical Agriculture and Food Security, Universiti Putra Malaysia (UPM), Serdang 43400, Selangor, Malaysia; cktan@moh.gov.my (C.T.); noorahya@upm.edu.my (N.N.J.); rashidah@upm.edu.my (R.S.); suganya@upm.edu.my (S.M.); 2 National Public Health Laboratory, Ministry of Health Malaysia, Lot 1853, Kampung Melayu Sungai Buloh, Sungai Buloh 47000, Selangor, Malaysia; 3 Department of Food Science, Faculty of Food Science and Technology, Universiti Putra Malaysia (UPM), Serdang 43400, Selangor, Malaysia; 4 Department of Pharmaceutical Chemistry, Faculty of Pharmacy, International Islamic University Malaysia, Kuantan 25200, Pahang, Malaysia; alfikhatib@iium.edu.my

**Keywords:** GC–MS, metabolomics, chicken, biomarkers, pectoralis major, serum, PCA

## Abstract

Globally, village chicken is popular and is known as a premium meat with a higher price. Food fraud can occur by selling other chicken breeds at a premium price in local markets. This study aimed to distinguish local village chicken from other chicken breeds available in the market, namely, colored broiler (Hubbard), broiler (Cobb), and spent laying hen (Dekalb) in pectoralis major and serum under commercial conditions using an untargeted metabolomics approach. Both pectoralis major and serum were analyzed using gas chromatography–mass spectrometry (GC–MS). The principal component analysis (PCA) results distinguished four different chicken breeds into three main groups for pectoralis major and serum. A total of 30 and 40 characteristic metabolites were identified for pectoralis major and serum, respectively. The four chicken breeds were characterized by the abundance of metabolites such as amino acids (L−glutamic acid, L−threonine, L−serine, L−leucine), organic acids (L−lactic acid, succinic acid, 3−hydroxybutyric acid), sugars (D−allose, D−glucose), sugar alcohols (myo−inositol), and fatty acids (linoleic acid). Our results suggest that an untargeted metabolomics approach using GC–MS and PCA could discriminate chicken breeds for pectoralis major and serum under commercial conditions. In this study, village chicken could only be distinguished from colored broiler (Hubbard) by serum samples.

## 1. Introduction

Chicken meat is known as the most popular poultry meat worldwide. The demand for poultry meat has increased in the past few decades due to healthy eating habits [1]; better choice and its lower cost than red meat; readiness for further processing; and it having no religious, cultural, or political prohibitions [2]. In the global poultry sector, including in Asian countries, village chickens have been shown significant demand for the past few decades, with prices two to four times higher than broiler [3]. Village chicken demand is notably high due to its uniqueness in flavor and better meat quality [4]. The market age for the village chicken is about 4–5 months, with 1–1.5 kg of live weight on average [5,6]. There are many occasions that underage colored broiler is sold as village chicken at a premium price to fraud the consumers for economic gain [7,8]. The manipulation of the market age of chickens sold in the market may lead to food fraud and counterfeiting risk due to the size similarity of the whole carcass between the chicken breeds. Furthermore, the consumers struggle to recognize the type of chicken breeds in terms of the retail cut of breast meat and meat products [9]. Although such food fraud may not threaten consumers’ health, a plausible solution to this problem is urgently needed to safeguard consumers’ rights and ensure fair trade. 

Metabolomics aims to study the low molecular weight of metabolites (<1000 Daltons) in an organism in tissues, biofluids, or cells. The obtained metabolite profiles could provide insight views of the metabolic responses towards the genetic or environmental variations [10]. There are two categories in metabolomics study, namely, targeted and untargeted. The targeted analysis is accessing the changes or responses on selected metabolites under the specifically defined conditions, mostly involving quantification. On the other hand, untargeted analysis is intended to detect as many metabolites as possible for trends or fingerprint determination without requiring compound quantification [11]. In metabolomics study, gas chromatography–mass spectrometry (GC–MS) is widely used due to its robustness, high sensitivity, and capability to provide a snapshot for a broad range of metabolites within a single analysis [12]. For instance, GC–MS coupled with multivariate statistical data analysis has been applied in previous reports for authentication of meat and seafood, such as beef of different geographic origins [13], cattle breeds, pork and chicken [14], chicken and pork mincemeat [15], different muscle types in fish [16], and mussel species [17]. 

In this study, we focused on four commercially available chicken breeds in the local market, namely, authentic village chicken, colored broiler (Hubbard), spent laying hen (Dekalb), and broiler (Cobb), which were obtained from the local supplier at the market age for untargeted metabolomics studies by using GC–MS. To the best of our knowledge, no official biomarkers or analytical protocols capable of distinguishing between chicken breeds are currently available. Therefore, this study aimed to explore an untargeted metabolomics study’s feasibility to distinguish village chicken from other chicken breeds commercially available in the local market. The information gained could serve as a proof-of-concept study for further investigation by the local authorities and industries.

## 2. Materials and Methods

### 2.1. Chemicals 

The analytical grade of solvents for methanol, chloroform, acetone, and hexane was purchased from Merck (Darmstadt, Germany). The pyridine was purchased from Sigma-Aldrich (Gillingham, UK). The derivatization reagents methoxylamine hydrochloride and *N*-methyl-*N*-(trimethylsilyl) trifluoroacetamide (MSTFA) were purchased from Acros Organics (Loughborough, UK). Ultrapure water was prepared by the Milli-Q system from Millipore Corporation (Billerica, MA, USA). All reference standards (arachidonic acid, cysteine, D−allose, D−glucose, L−phenylalanine, linoleic acid, L−aspartic acid, L−leucine, L−valine, stearic acid, urea, and fumaric acid) and internal standards (2−isopropylmalic acid, L−norleucine, ribitol, and heptadecanoic acid) were purchased from Sigma−Aldrich (St. Louis, MO, USA).

### 2.2. Sample Collection and Ethical Approval

Four commercially major chicken breeds, namely, market age of local authentic village chicken, VC (14 weeks, 1010.5 ± 47.6 g); broiler Cobb, BC (6 weeks, 2487.5 ± 53.5 g); colored broiler Hubbard, BH (10 weeks, 2055.5 ± 66.4 g); and spent laying hen Dekalb, LD (72 weeks, 1847.9 ± 58.7 g) were used in this study. Samples were all females and purchased directly from local suppliers, which have been confirmed in producing the respective chicken breeds. The commercial feed fed to all chicken breeds at the respective commercial farms was the same brand (Gold Coin) with similar ingredients, i.e., corn and soybean. At the commercial farms, BC was reared at closed housing floor system, VC and BH were reared at open house floor system, and LD was reared at closed housing cages system. The stocking density with the floor system for BC, VC, and BH was approximately eight birds per m^2^. For LD, the stocking density with the cages system was approximately 11 birds per m^2^. All birds were fed ad libitum with commercial feed and water until eight hours before slaughter. In short, all birds were slaughtered by cutting of jugular veins and rested for approximately two minutes, followed by manual de-feathering and de-skinning. The pectoralis major and serum were collected for five individuals from each chicken breed. Pectoralis major muscles were snap-frozen using liquid nitrogen and stored at −80 °C until further analysis. Blood samples were collected during bleeding and kept for 15 min at room temperature until coagulation. After centrifugation (5810R, Eppendorf AG, Hamburg, Germany) at 4000× *g* for 10 min, sera were aliquoted and stored at −80 °C until further analysis [18]. Information about chicken breeds used in this study is summarized in Table S1. Photos of four chicken breeds and samples collected (pectoralis major and chicken serum) are displayed in Figures S1 and S2. 

All the animal use and experimental procedures performed in this study were evaluated and approved by the Institutional Animal Care and Use Committee (IACUC), Universiti Putra Malaysia (UPM), with the approval number: UPM/IACUC/AUP-R022/2019.

### 2.3. Preparation of Muscle Samples for GC–MS Analysis

The muscle sample preparation for GC–MS analysis was performed on the basis of the work of Mabuchi et al. [19] with slight modification. In brief, 250 mg of freeze-dried powdered samples were added with 6 µL of 2−isopropylmalic acid (1.0 mg/mL) and homogenized with a mixed solution of methanol/ultrapure water/chloroform (2.5/1/1 *v*/*v*/*v*, 1 mL) for 5 min. After centrifugation (16,000× *g*, 0 °C, 5 min), 400 µL ultrapure water was added to 800 µL of the supernatant and vortexed for 1 min, followed by centrifugation (16,000× *g*, 0 °C, 5 min). A total of 600 µL of the supernatant was completely dried with a nitrogen gas stream. For the two-step derivatization, 50 µL of pyridine containing 20 mg/mL methoxyamine hydrochloride was added to the sample and incubated with a thermomixer (Thermomixer Comfort, Eppendorf AG, Hamburg, Germany) for 90 min at 30 °C. Next, 100 µL of *N*-methyl-*N*-(trimethylsilyl) trifluoroacetamide was further added and incubated for 30 min at 37 °C. The derivatized samples were centrifuged at room temperature (16,000× *g*, 5 min). A total of 80 µL of the supernatant was transferred to a sample vial and submitted to GC–MS analysis.

### 2.4. Preparation of Serum Samples for GC–MS Analysis

The serum sample preparation for GC–MS was performed on the basis of Trimigno et al. [20] with slight modification. In brief, 100 µL of serum samples was added with 6 µL of 2-isopropylmalic acid (1.0 mg/mL) and vortexed with 300 µL cool methanol for 5 min. After centrifugation (15,800× *g*, 0 °C, 15 min), 370 µL of the supernatant was dried completely with a nitrogen gas stream. For the two-step derivatization, 50 µL of pyridine containing 20 mg/mL methoxyamine hydrochloride was added to the sample, vortexed for 15 s, and incubated with a thermomixer for 15 min at 80 °C. Next, 50 µL of *N*-methyl-*N*-(trimethylsilyl) trifluoroacetamide was further added and incubated for 15 min at 80 °C. The derivatized samples were cooled to room temperature and centrifuged (15,800× *g*, 15 min). A total of 80 µL of the supernatant was transferred to a sample vial and submitted to GC–MS analysis.

### 2.5. Preparation of Quality Control (QC) Samples

Five grams of all individual freeze-dried pectoralis major samples were pooled and combined as the QC sample. On the other hand, for serum, 5 mL of all individual samples were pooled and combined as the QC sample. Both QC samples of pectoralis major and serum were stored at −80 °C until further analysis. These QC samples were analyzed together with the samples in each analytical batch, as described in Section 2.3 and Section 2.4. Moreover, to verify the GC–MS condition on precision and retention time before this study was performed, we injected the extracted QC sample for pectoralis major five times. The relative standard deviation (RSD) was calculated on each metabolite, as displayed in Table S2.

### 2.6. Preparation of Standard Solution

The standard solutions preparation was carried out according to Mykhailenko et al. [21] with some modifications. One milligram of each reference standard and internal standard were diluted using deionized water, acetone, or ethanol. The standard solutions were mixed accordingly at different concentration levels and dried under nitrogen gas stream, followed by the derivatization procedures for pectoralis major and serum as described in Section 2.3 and Section 2.4.

### 2.7. GC–MS Analysis

GC–MS analysis was performed following the method described by Grabež et al. [22] with some modification. One microliter of derivatized samples were analyzed on an Agilent 7890B GC system connected with an Agilent 7000C MS detector. An HP-5MS capillary column (i.d. 30 m × 0.25 mm, firm thickness 0.25 µm) was used for the separation. The carrier gas (He) flow rate through the column was 1 mL/min. The GC temperature program was as follows: 80 °C for 5 min, ramped at 4 °C/min until 315 °C. The analysis time was 83 min. Mass scans were conducted in full scan mode for the range *m*/*z* 35 to 600. Electron ionization was performed with 70 eV. The mass spectra of chromatograms were processed using Masshunter software and further compared with the National Institute of Standards and Technology (NIST) 14 mass spectral library. The consideration is only for those metabolites that achieved >90% matching probability score [23].

### 2.8. GC–MS Data Processing

After GC–MS analysis, data pre-processing was performed on the basis of the work of Murugesu et al. [24]. In short, for each raw chromatogram obtained from GC–MS analysis, the conversion to cdf format was carried out using ACD/Spec Manager v.12.00 (Advanced Chemistry Development, Inc., ACD/Labs Ontario, Toronto, ON, Canada). Next, all data in cdf format were further processed using MZmine software (version 2.3, Okinawa Institute of Science and Technology Graduate University, Kunigami-gun, Okinawa, Japan) with steps as follows: systematic noise filtering, automatic peak detection, baseline correction, data binning, deconvolution, and chromatographic alignment. All the processed data were summarized in Excel sheet form and subjected to multivariate data analysis (MVDA). PCA score scatter plots, loading plots, and loading column plots were generated using SIMCA P^+^14.0 software (version 14.0, Umetrics, Umeå, Västerbotten, Sweden). 

### 2.9. Statistical Analysis

For the quantification results with 12 commercial standards, we performed one-way ANOVA analysis with Tukey’s multiple comparison test using Minitab 18 (Minitab Inc., State College, PA, USA). For the semi-quantification results, a two-sample *t*-test was performed using Minitab 18 to determine the significant difference between chicken breeds from two different clusters. The value of *p* < 0.05 for both statistical analyses was considered significant.

### 2.10. Method Validation of Extraction Protocols

Method validation was conducted with 12 reference standards according to ISO 5725 for limit of detection (LOD), limit of quantification (LOQ), linearity, precision, and accuracy. Twelve reference standards were selected on the basis of the main classes detected in the chicken samples for pectoralis major muscle and serum, including organic acid, amino acid, fatty acid, and sugars. The calibration curve was established at six concentration levels for each reference standard, and correlation coefficients (R^2^) were calculated. The LOD and LOQ were calculated on the basis of the signal-to-noise ratio of 3 times and 10 times, respectively. The regression model test, lack of fit test, and significance test were performed using the LINEST function in Excel for the linearity confirmation of each compound’s calibration curve. The precision and accuracy of the method were performed at three different concentrations for pectoralis major and serum sample extracts, with six injections and two replicates. 

### 2.11. Quantification of Metabolites in Pectoralis Major and Serum

Quantification of 12 targeted metabolites was carried out according to the methods described in Mykhailenko et al. [21], Murugesu et al. [24], and Shi et al. [25] with some modifications. The limit of quantification (LOQ), the limit of detection (LOD), linearity, precision, and trueness were determined using reference standards. The amount for each targeted metabolite was calculated via substitution of the peak area into the regression equation generated from its reference standard curve. For other identified metabolites, the ratio between the peak area of the metabolite and 2−isopropylmalic acid was calculated for semi-quantification. 

### 2.12. Calculation of the Recovery of the Internal Standards

The recovery of the internal standard in pectoralis major and serum samples was carried out according to the work of Murugesu et al. [24] with some modifications. Three internal standards were purchased, namely, L−norleucine, ribitol, and heptadecanoic acid. In short, five concentration levels of each internal standard solution were prepared, followed by the same derivatization procedure for pectoralis major and serum samples. For the recovery calculation, the 250 mg pectoralis major and 100 µL serum samples were added with an amount of each internal standard solution (R_1_). The samples were extracted and derivatized according to Section 2.3 and Section 2.4. The recovered amount (R_2_) of each internal standard was calculated. The recovery, R, was determined with the following equation:

R (%) = (R_2_/R_1_) × 100% (1)

## 3. Results

In this result section, the GC–MS results and data processing are described in Section 3.1. The multivariate data analysis (MVDA) for differentiation of four chicken breeds is described in Section 3.2. Method performances of the method validation on two extraction protocols for pectoralis major and serum are reported in Section 3.3. Sample quantification and semi-quantification results are shown in Section 3.4. Moreover, the additional information for the proximate analysis results on the feed samples is reported in Section 3.5.

### 3.1. GC–MS Chromatogram of Pectoralis Major and Serum

In the present study, 49 and 62 annotated metabolites were observed from GC–MS total ion chromatogram (TIC) for pectoralis major and serum, respectively, as summarized in Table 1 and Table 2. The list of annotated metabolites for pectoralis major and serum is shown in Tables S3 and S4. TIC for pectoralis major and serum of four chicken breeds are displayed in Figures S3 and S4. The metabolites identified for both pectoralis major and serum were mainly composed of amino acids, fatty acids, organic acids, sugars, and others. Nonetheless, no great differences in the metabolite classification were observed for pectoralis major and serum between the chicken breeds. All tentatively identified metabolites in pectoralis major and serum were considered with >90% matching probability score from NIST 14 mass spectral library in GC–MS and further analyzed with MVDA [23,26].

### 3.2. Multivariate Data Analysis

**Figure 1:**
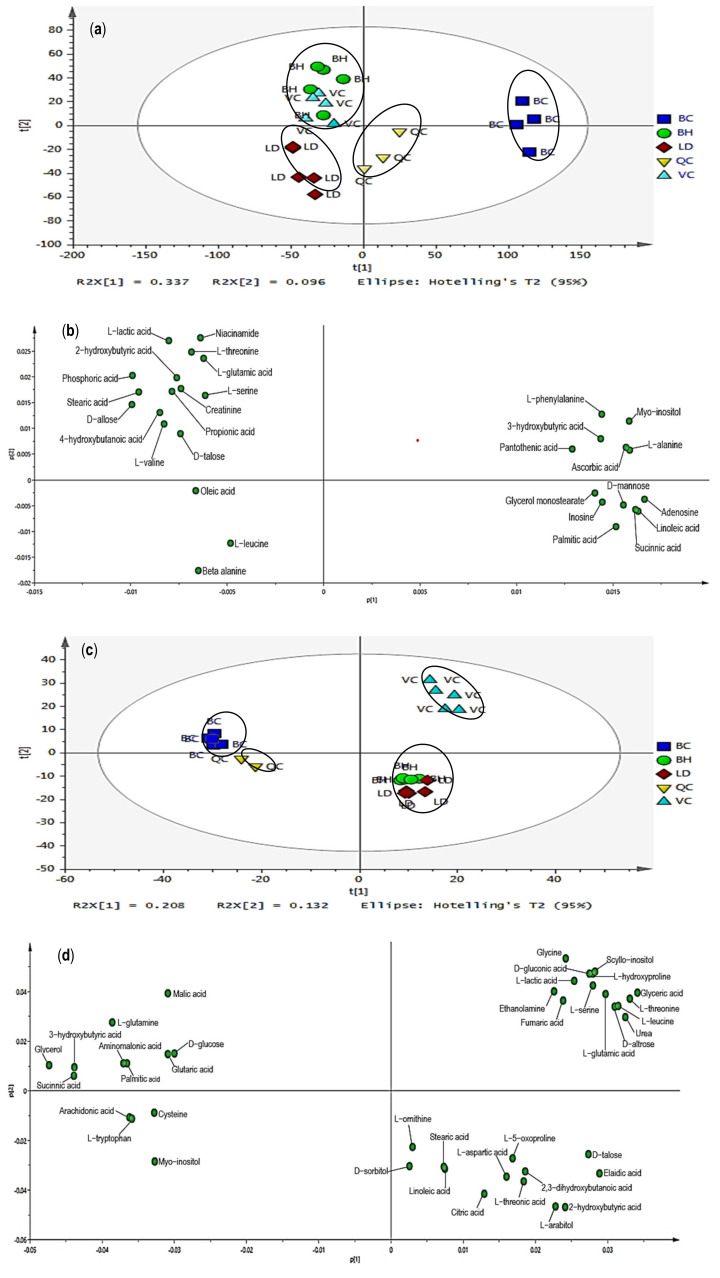
PCA score scatter and loading plots for pectoralis major and serum between different chicken breeds. (**a**,**c**) PCA score scatter plots displaying cluster groups in pectoralis major and serum, respectively. (**b**,**d**) PCA loading plots displaying the metabolites contributed to the group clustering in pectoralis major and serum, respectively.

**Figure 2:**
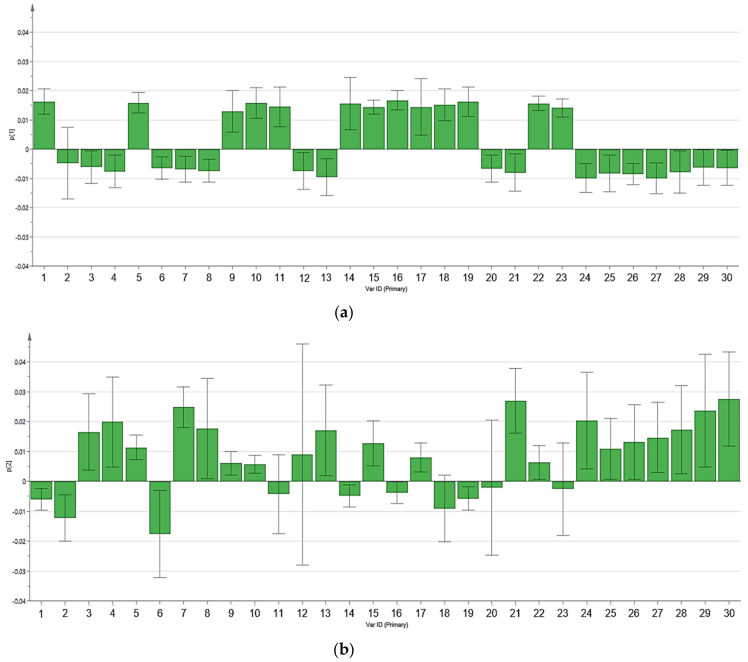
PCA loading column plots with jack-knife uncertainty bars for pectoralis major of different chicken breeds. (**a**,**b**) PCA loading column plots displaying the correlation of metabolites in PCA loading plots on the basis of principal components 1 and 2 in score scatter plots, respectively. Assignments: 1. linoleic acid, 2. beta-alanine, 3. L-serine, 4. 2-hydroxybutric acid, 5. myo-inositol, 6. L-leucine, 7. L-threonine, 8. creatinine, 9. pantothenic acid, 10. L-alanine, 11. inosine, 12. D-talose, 13. stearic acid, 14. D-mannose, 15. L-phenylalanine, 16. adenosine, 17. 3-hydroxybutyric acid, 18. palmitic acid, 19. succinic acid, 20. oleic acid, 21. L-lactic acid, 22. ascorbic acid, 23. glycerol monostearate, 24. phosphoric acid, 25. L-valine, 26. 4-hydroxybutanoic acid, 27. D-allose, 28. propionic acid, 29. L-glutamic acid, 30. niacinamide.

**Figure 3:**
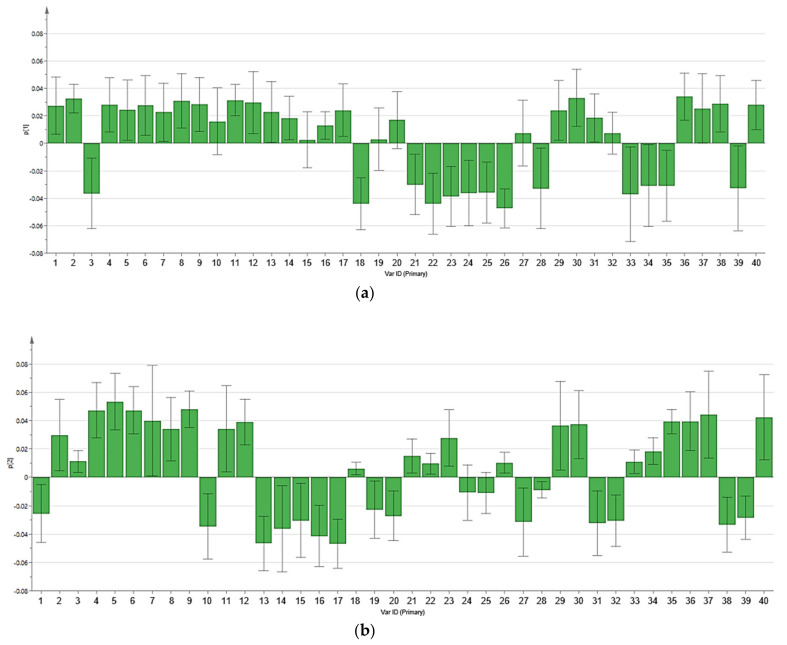
PCA loading column plots with jack-knife uncertainty bars for chicken serum of different chicken breeds. (**a**,**b**) PCA loading column plots displaying the correlation of metabolites in PCA loading plots on the basis of principal components 1 and 2 in score scatter plots, respectively. Assignments: 1. D-talose, 2. urea, 3. palmitic acid, 4. L-hydroxyproline, 5. glycine, 6. D-gluconic acid, 7. ethanolamine, 8. D-altrose, 9. scyllo-inositol, 10. L-aspartic acid, 11. L-leucine, 12. L-glutamic acid, 13. L-arabitol, 14. L-threonic acid, 15. D-sorbitol, 16. citric acid, 17. 2-hydroxybutyric acid, 18. succinic acid, 19. L-ornithine, 20. L-5-oxoproline, 21. D-glucose, 22. 3-hydroxybutyric acid, 23. L-glutamine, 24. arachidonic acid, 25. L-tryptophan, 26. glycerol, 27. linoleic acid, 28. cysteine, 29. fumaric acid, 30. L-threonine, 31. 2,3-dihydroxybutanoic acid, 32. stearic acid, 33. aminomalonic acid, 34. glutaric acid, 35. malic acid, 36. glyceric acid, 37. L-lactic acid, 38. Elaidic acid, 39. Myo-inositol, 40. L-serine.

Principal component analysis (PCA) was performed to provide overall distribution and differences of metabolomic profiles between four chicken breeds, as displayed in Figure 1. Score scatter plots (Figure 1a,c) showed the differentiation among four chicken breeds, while loading plots (Figure 1b,d) explained the metabolites responsible for the chicken breed differentiation in score scatter plots. Multivariate data analysis through PCA of this study has been validated with the summary of fit analysis. The difference between R^2^X and Q^2^ values in the summary of fit was not larger than 0.3, indicating the validity of this model [27]. In score scatter plots, four chicken breeds were differentiated into three distinct groups on the basis of the metabolomic profiles for pectoralis major and serum. In Figure 1a, for pectoralis major samples, BC and LD were clustered separately at the right and lower left of the ellipse, respectively. Nonetheless, both VC and BH were clustered together at the upper left of the ellipse and could not be differentiated. On the other hand, Figure 1c for chicken serum samples showed that VC and BC were clearly separated at the upper right and upper left of the ellipse, respectively. Meanwhile, both BH and LD serum samples were clustered together at the lower right of the ellipse and could not be differentiated. Loading plots (Figure 1b,d) illustrated the metabolites involvement for the differentiation of four chicken breeds in score scatter plots. In addition, the PCA loading column plots with jack-knife uncertainty bars (Figure 2 and Figure 3) further showed the correlation of the individual metabolite correspond to the chicken breeds clustering in score scatter plots. The loading column plots with the jack-knife error bar not passing zero (0) indicated the significant correlation of respective metabolites to the differentiation of chicken breeds [27]. The tentative metabolites contributing to the clustering of different chicken breeds for pectoralis major and serum are displayed in Table 3 and Table 4, respectively. On the basis of the principal component (PC) two in Figure 1b, we see that several metabolites were located in the positive direction, including metabolites of amino acids (L−threonine, L−serine, L−glutamic acid), organic acids (L−lactic acid, 2−hydroxybutyric acid), vitamins (niacinamide), fatty acids (stearic acid, 4−hydroxybutanoic acid), and others (creatinine, phosphoric acid, propionic acid). These metabolites showed a positive correlation with the cluster of VC and BH. Meanwhile, metabolic components located in the negative direction were correlated with BC and LD clusters (i.e., beta−alanine, D−mannose, linoleic acid, and succinic acid). On the other hand, on the basis of both PC1 and PC2 in Figure 1d, we see that 14 metabolites that were located in the positive direction showed a positive correlation to the VC cluster for serum, including metabolites of the amino acid (glycine, L−glutamic acid, L−threonine, L−serine, L−leucine, L−hydroxyproline), sugars (D-altrose), organic acids (L−lactic acid, fumaric acid), sugar acids and sugar alcohols (D−gluconic acid, glyceric acid, scyllo−inositol), and others (urea, ethanolamine). Moreover, on the basis of the PC1, we see that the BC cluster was constituted of several metabolites such as arachidonic acid, cysteine, D−glucose, glycerol, myo−inositol, succinic acid, and 3−hydroxybutyric acid. Meanwhile, BH and LD clusters were constituted of several other metabolites (i.e., citric acid, L−arabitol, L−aspartic acid, and 2−hydroxybutyric acid) on the basis of the PC2. 

### 3.3. Reliability of the Extraction Protocols

The extraction protocol for both pectoralis major muscle and serum was validated and evaluated using 12 reference standards. The limit of detection (LOD), the limit of quantitation (LOQ), linearity, precision, and accuracy were measured and presented in Tables S5 and S6. The LOD and LOQ for pectoralis major muscle were 1.9–7.5 µg/mg and 8–25 µg/mg, respectively. The LOD and LOQ for serum were 1.5–138.4 µg/mL and 5–500 µg/mL, respectively. Moreover, all the established calibration curves achieved good linearity with correlation coefficients (R^2^) > 0.99. The recovery of all measured compounds was reported as >90%. The relative standard deviation of repeatability (RSDr) at three different concentrations was reported in the range of 0.36–1.49%.

### 3.4. Quantification and Semi-Quantification of Samples

For both pectoralis major and serum matrices, the content of 12 selected metabolites contributing to the group clustering was determined. One-way ANOVA analysis was performed to determine the significant difference between four chicken breeds for the metabolites as displayed in Table 5 and Table 6. Moreover, the semi-quantification values of other characteristic metabolites were compared for any two breeds of VC, BC, BH, and LD, on the basis of the clusters derived in the PCA for pectoralis major and serum, as displayed in Tables S7 and S8.

### 3.5. Proximate Analysis of Commercial Feed Samples

Proximate analysis, including the ash, moisture content, crude fiber, crude protein, and crude fat, were performed for the chicken feeds, as displayed in Table S9.

## 4. Discussion

In the present study, MVDA through PCA was employed to provide an overview and visualize the differences in metabolomic profiles between the chicken breeds at their respective market age. In previous studies, PCA has been successfully applied for distinguishing different species, breeds, tissues, and biofluids [26,28,29]. In PCA score scatter plots for pectoralis major and serum (Figure 1a,c), the four chicken breeds were differentiated into three clusters for both matrices. MVDA results showed that VC and the other three chicken breeds were characterized by amino acids, organic acids, fatty acids, sugars, sugar alcohols, vitamins, and others in loading plots, as displayed in Figure 1b,d. Contrary to expectation, VC and BH breeds could not be distinguished by pectoralis major. The possible explanation is that these two breeds may share similar metabolite profiles as both breeds are classified as slow-growing chickens [5,30]. Nonetheless, VC was successfully distinguished from the other three chicken breeds by serum. This finding may indicate that serum is a better choice to differentiate VC from other breeds on the basis of GC–MS analytical platform. In this study, the four chicken breeds were obtained from local commercial farms on the basis of birds’ market age, representing the actual condition in the local market. The difference in market ages between the chicken breeds may possess different metabolism, contributing to the different metabolic profiles between the chicken breeds [31]. Nonetheless, all the chicken breeds in the present study were fed with the same brand of commercial feeds (Gold Coin) at their respective commercial farm and reared under similar standard conditions.

A total of 30 and 40 characteristic metabolites, which can be essentially applied for the differentiation of the chicken breeds, were tentatively identified for pectoralis major and serum, respectively, as displayed in Table 3 and Table 4. In the present study, several potential biomarkers such as L-glutamic acid, L-lactic acid, L-threonine, and L-serine contributed to both clusters involving VC for pectoralis major and serum. It is also worth noting that another three metabolites, namely, myo-inositol, 3-hydroxybutyric acid, and succinic acid, contributed to the BC cluster for both pectoralis major and serum. The MVDA, through PCA, pinpoints the biomarkers, which are significant to the respective chicken breed in terms of the number of metabolites in each chicken breed. These biomarkers are present in the chicken breeds at different amounts. Therefore, the contribution of all characteristic metabolites for chicken breeds clustering in score scatter plots were further confirmed by quantification with commercial standards or semi-quantification, as reported in Section 3.4.

In this study, the VC cluster for serum was characterized by several important metabolites, including fumaric acid and urea. The amount of both metabolites was further confirmed and quantified using commercial standards, as displayed in Table 6. We found that fumaric acid was only quantified in VC and not detected (below LOQ) for other chicken breeds (BC, BH, LD). Moreover, the amount of urea in serum was the highest in VC, followed by LD, BC, and BH. According to the European Food Safety Authority (EFSA), no accumulation of fumaric acid in animal tissues and related products is expected for its exogenous form derived from the diet [32]. On the other hand, urea is an end product of protein metabolism in poultry [33]. According to Donsbough et al. [34], urea and uric acid content in broilers’ blood could reflect the amino acid utilization in birds. Therefore, fumaric acid and urea may be considered as potential distinguishing biomarkers for VC in serum.

Interestingly, for pectoralis major, the VC cluster was characterized by both L-lactic acid and creatinine. Moreover, the VC cluster for serum was also characterized by L-lactic acid. A previous study by Kong et al. [35] reported that lactic acid and succinic acid were detected in the chicken soup and chicken hydrolysate of China native chicken (Sanhuang), with lactic acid being the most prevalent. Mabuchi et al. [19] reported that lactic acid and creatinine accumulation are related to exercise and contributed to fish species of *Pagrus major*, which demonstrated more active behavior compared to other fish species. Therefore, the nature of indigenous chicken, which is more physically active with higher locomotor activities, could explain this finding [36]. On the basis of the semi-quantification results reported in Tables S7 and S8, L-lactic acid in VC for both pectoralis major and serum was found to be approximately 5- and 3-fold higher than other chicken breeds in different clusters, respectively. Moreover, creatinine content in VC was found to be 3- to 10-fold higher than other breeds in pectoralis major. These results were consistent with the contribution of both metabolites in loading plots, indicating the potential of these metabolites in distinguishing VC from other chicken breeds.

Several amino acids that contributed to the differentiation of chicken breeds in score scatter plots were identified in loading plots. VC for both pectoralis major and serum matrices were characterized by L-glutamic acid, L-threonine, and L-serine. Moreover, MVDA results also showed that L-leucine, glycine, and L-hydroxyproline characterized VC for serum. For instance, L-hydroxyproline is a non-essential amino acid that plays an essential role in the synthesis of glycine. Glycine is known as an essential amino acid present in chicken [37]. In this study, the quantification and semi-quantification results showed that these amino acids’ content in VC was significantly higher than other chicken breeds. In previous studies, indigenous/native chickens of Thailand and Bali, Indonesia, were reported to have a high content of glutamic acid [38,39]. Moreover, slow-growing chicken breeds were also reported to have higher protein content and better amino acid profiles than fast-growing broilers [36]. Therefore, these amino acids are potential biomarkers to distinguish VC from other chicken breeds. Nonetheless, some studies have investigated the synergistic effects of dietary branched-chain amino acids (BCAA) supplementation, including leucine, valine, and isoleucine in poultry diets [40,41]. Taken together, BCAA for L-leucine and L-valine, which were identified as characteristic metabolites in this study, may need further confirmation studies. On the other hand, MVDA results showed that the BC cluster for serum was characterized by cysteine. Cysteine is a non-essential sulfur amino acid that could be synthesized from the degradation of L-methionine via the transsulfuration pathway in birds. The quantification result showed that the amount of cysteine was only quantified in the serum of BC and not detected (below LOQ) for the other three chicken breeds. This result indicated that cysteine is a potential biomarker to distinguish BC from other chicken breeds.

In the present study, it is worth noting that another three important metabolites, namely, myo-inositol, glucose, and glycerol contributed to the BC cluster. Loading plots (Figure 1b,d) showed that myo-inositol contributed to the BC cluster for pectoralis major and serum. Moreover, glucose and glycerol, which are associated with glucose metabolism, contributed to the BC cluster for serum. Myo-inositol is a sugar alcohol naturally found in grains, nuts, beans, and fruits [42]. For instance, the commercial feed consisting of corn, soybean, and other grains used in poultry industries may contribute to the myo-inositol formation in poultry [43]. A previous study by Cowieson et al. [44] reported that myo-inositol supplementation in diet could increase glucose, insulin, and glucagon content in the blood plasma. In this study, quantification results showed that the glucose content in serum was the highest for BC, followed by VC, LD, and BH. Concomitantly, glycerol, which plays an essential role as the substrate in glucose production in animals, also contributed to the BC cluster in serum [45]. Thus, these three metabolites could reflect the glucose metabolism in the BC breed and utilize as effective biomarkers for BC.

## 5. Conclusions

The present study demonstrated the differentiation of local authentic village chicken from other three chicken breeds at their respective market age by an untargeted metabolomics approach using GC–MS. MVDA through PCA showed that four chicken breeds were successfully distinguished into three different clusters for pectoralis major and chicken serum. In the score scatter plot for serum, VC was successfully distinguished from another three chicken breeds. On the other hand, both VC and BH were mixed in one cluster for pectoralis major and not separated. A total of 30 and 40 characteristic metabolites, including amino acids, organic acids, fatty acids, sugars, sugar alcohols, and others, were identified for both pectoralis major and serum, respectively. These metabolites are effective biomarkers that are applicable to distinguish the market age of local authentic village chicken from other chicken breeds in the local market. The results from this study serve as baseline data, representing the actual condition in the local market, which can be applied for the authentication of market age and size of local authentic village chicken by regulatory agencies.

## Figures and Tables

**Table 1:** The number of annotated metabolites in pectoralis major of different chicken breeds.

Classification	Authentic Village Chicken	Broiler (Cobb)	Colored Broiler (Hubbard)	Spent Laying Hen (Dekalb)
Amino acid	9 (25.7)	10 (23.8)	7 (23.3)	10 (27.8)
Fatty acid	7 (20.0)	9 (21.4)	6 (20.0)	9 (25.0)
Organic acid	6 (17.1)	5 (11.9)	4 (13.3)	5 (13.9)
Sugar	3 (8.6)	4 (9.5)	4 (13.3)	4 (11.1)
Sugar alcohol	2 (5.7)	2 (4.8)	1 (3.3)	1 (2.8)
Vitamin	1 (2.9)	3 (7.1)	2 (6.7)	1 (2.8)
Other	7 (20.0)	9 (21.4)	6 (20.0)	6 (16.7)
Total	35 (100)	42 (100)	30 (100)	36 (100)

Number in parentheses indicates the percentage of each metabolite class for each chicken breed.

**Table 2:** The number of annotated metabolites in serum of different chicken breeds.

Classification	Authentic Village Chicken	Broiler (Cobb)	Colored Broiler (Hubbard)	Spent Laying Hen (Dekalb)
Amino acid	14 (28.6)	19 (36.5)	14 (34.1)	12 (27.9)
Fatty acid	5 (10.2)	5 (9.6)	7 (17.1)	6 (14.0)
Organic acid	10 (20.4)	10 (19.2)	6 (14.6)	9 (20.9)
Sugar	5 (10.2)	5 (9.6)	4 (9.8)	5 (11.6)
Sugar alcohol	6 (12.2)	6 (11.5)	4 (9.8)	6 (14.0)
Other	9 (18.4)	7 (13.5)	6 (14.6)	5 (11.6)
Total	49 (100)	52 (100)	41 (100)	43 (100)

Number in parentheses indicates the percentage of each metabolite class for each chicken breed.

**Table 3:** Tentative untargeted metabolites for pectoralis major contributed to the group clustering in PCA score scatter plot.

No.	RT (min)	Tentative Metabolites	Molecular Weight (g/mol)	Derivatized Molecular Weight (g/mol)	Probability (%)	Molecular Formula	Derivatized Molecular Formula
1	7.005	2−hydroxybutyric acid	104.10	248.13	92.87	C_4_H_8_O_3_	C_10_H_24_O_3_Si_2_
2	7.892	3−hydroxybutyric acid	104.10	248.13	93.39	C_4_H_8_O_3_	C_10_H_24_O_3_Si_2_
3	10.201	4−hydroxybutanoic acid	104.10	248.13	95.20	C_4_H_8_O_3_	C_10_H_24_O_3_Si_2_
4	55.193	Adenosine	267.24	555.26	92.54	C_10_H_13_N_5_O_4_	C_22_H_45_N_5_O_4_Si_4_
5	36.315	Ascorbic acid	176.12	464.19	95.73	C_6_H_8_O_6_	C_18_H_40_O_6_Si_4_
6	8.743	Beta-alanine	89.09	233.13	92.87	C_3_H_7_NO_2_	C_9_H_23_NO_2_Si_2_
7	22.055	Creatinine	113.12	329.66	92.16	C_4_H_7_N_3_O	C_13_H_31_N_3_OSi_3_
8	35.148	D−talose	180.16	569.29	91.78	C_6_H_12_O_6_	C_22_H_55_NO_6_Si_5_
9	35.616	D−allose	180.16	569.29	91.64	C_6_H_12_O_6_	C_22_H_55_NO_6_Si_5_
10	34.772	D−mannose	180.16	540.26	94.32	C_6_H_12_O_6_	C_21_H_52_O_6_Si_5_
11	58.382	Glycerol monostearate	358.56	502.39	95.93	C_21_H_42_O_4_	C_27_H_58_O_4_Si_2_
12	53.819	Inosine	268.23	556.24	93.76	C_10_H_12_N_4_O_5_	C_22_H_44_N_4_O_5_Si_4_
13	5.62	L−lactic acid	90.08	234.11	95.64	C_3_H_6_O_3_	C_9_H_22_O_3_Si_2_
14	6.361	L−alanine	89.09	233.13	90.94	C_3_H_7_NO_2_	C_9_H_23_NO_2_Si_2_
15	24.789	L−glutamic acid	147.13	363.17	91.10	C_5_H_9_NO_4_	C_14_H_33_NO_4_Si_3_
16	7.643	L−leucine	131.17	203.13	94.27	C_6_H_13_NO_2_	C9H21NO2Si
17	24.576	L−phenylalanine	165.19	309.16	91.11	C_9_H_11_NO_2_	C_15_H_27_NO_2_Si_2_
18	10.942	L−serine	105.09	249.12	94.74	C_3_H_7_NO_3_	C_9_H_23_NO_3_Si_2_
19	16.046	L−threonine	119.12	335.18	91.59	C_4_H_9_NO_3_	C_13_H_33_NO_3_Si_3_
20	9.63	L−valine	117.15	261.16	92.04	C_5_H_11_NO_2_	C_11_H_27_NO_2_Si_2_
21	43.301	Linoleic acid	280.45	352.28	98.84	C_18_H_32_O_2_	C21H40O2Si
22	40.847	Myo-inositol	180.16	612.30	91.98	C_6_H_12_O_6_	C_24_H_60_O_6_Si_6_
23	18.81	Niacinamide	122.12	194.09	91.08	C_6_H_6_N_2_O	C_9_H_14_N_2_OSi
24	43.472	Oleic acid	282.46	354.30	91.88	C_18_H_34_O_2_	C_21_H_42_O_2_Si
25	38.502	Palmitic acid	256.42	328.28	93.17	C_16_H_32_O_2_	C_19_H_40_O_2_Si
26	37.390	Pantothenic acid	219.23	435.23	90.40	C_9_H_17_NO_5_	C_18_H_41_NO_5_Si_3_
27	30.373	Phosphoric acid	98.00	460.17	96.31	H_3_PO_4_	C_15_H_41_O_6_PSi_4_
28	31.873	Propionic acid	74.08	474.15	92.78	C_3_H_6_O_2_	C_15_H_39_O_7_PSi_4_
29	44.286	Stearic acid	284.48	356.31	94.06	C_18_H_36_O_2_	C_21_H_44_O_2_Si
30	12.972	Succinic acid	118.09	262.11	98.30	C_4_H_6_O_4_	C_10_H_22_O_4_Si_2_

**Table 4:** Tentative untargeted metabolites for chicken serum contributing to the group clustering in PCA score scatter plot.

No.	RT (min)	Tentative Metabolites	Molecular Weight (g/mol)	Derivatized Molecular Weight (g/mol)	Probability (%)	Molecular Formula	Derivatized Molecular Formula
1	14.758	2,3-dihydroxybutanoic acid	120.10	336.16	91.07	C_4_H_8_O_4_	C_13_H_32_O_4_Si_3_
2	6.932	2-hydroxybutyric acid	104.10	248.13	94.29	C_4_H_8_O_3_	C_10_H_24_O_3_Si_2_
3	7.856	3-hydroxybutyric acid	104.10	248.13	96.23	C_4_H_8_O_3_	C_10_H_24_O_3_Si_2_
4	19.059	Aminomalonic acid	119.08	335.14	93.30	C_3_H_5_NO_4_	C_12_H_29_NO_4_Si_3_
5	47.559	Arachidonic acid	304.47	376.28	94.80	C_20_H_32_O_2_	C_23_H_40_O_2_Si
6	32.129	Citric acid	192.12	480.19	91.79	C_6_H_8_O_7_	C_18_H_40_O_7_Si_4_
7	22.201	Cysteine	121.16	337.14	96.55	C_3_H_7_NO_2_S	C_12_H_31_NO_2_SSi_3_
8	37.986	D-altrose	180.16	540.26	95.21	C_6_H_12_O_6_	C_21_H_52_O_6_Si_5_
9	37.117	D-gluconic acid	196.16	628.30	95.51	C_6_H_12_O_7_	C_24_H_60_O_7_Si_6_
10	35.36	D-glucose	180.16	569.29	94.39	C_6_H_12_O_6_	C_22_H_55_NO_6_Si_5_
11	36.102	D-sorbitol	182.17	614.32	92.80	C_6_H_14_O_6_	C_24_H_62_O_6_Si_6_
12	34.808	D-talose	180.16	540.26	95.81	C_6_H_12_O_6_	C_21_H_52_O_6_Si_5_
13	43.442	Elaidic acid	282.46	354.30	95.21	C_18_H_34_O_2_	C_21_H_42_O_2_Si
14	11.246	Ethanolamine	61.08	277.17	93.99	C_2_H_7_NO	C_11_H_31_NOSi_3_
15	14.114	Fumaric acid	116.07	260.09	93.08	C_4_H_4_O_4_	C_10_H_20_O_4_Si_2_
16	23.13	Glutaric acid	132.11	319.13	90.47	C_5_H_8_O_4_	C_12_H_25_NO_5_Si_2_
17	13.846	Glyceric acid	106.08	322.15	91.52	C_3_H_6_O_4_	C_12_H_30_O_4_Si_3_
18	11.781	Glycerol	92.09	308.17	91.88	C_3_H_8_O_3_	C_12_H_32_O_3_Si_3_
19	6.683	Glycine	75.07	219.11	92.33	C_2_H_5_NO_2_	C_8_H_21_NO_2_Si_2_
20	28.555	L-arabitol	152.15	512.27	91.68	C_5_H_12_O_5_	C_20_H_52_O_5_Si_5_
21	21.07	L-aspartic acid	133.10	349.16	92.53	C_4_H_7_NO_4_	C_13_H_31_NO_4_Si_3_
22	24.728	L-glutamic acid	147.13	363.17	92.22	C_5_H_9_NO_4_	C_14_H_33_NO_4_Si_3_
23	30.172	L-glutamine	146.19	362.19	91.72	C_5_H_10_N_2_O_3_	C_14_H_34_N_2_O_3_Si_3_
24	21.198	L-hydroxyproline	131.13	347.18	90.40	C_5_H_9_NO_3_	C_14_H_33_NO_3_Si_3_
25	5.389	L-lactic acid	90.08	234.11	96.28	C_3_H_6_O_3_	C_9_H_22_O_3_Si_2_
26	7.576	L-leucine	131.17	203.13	94.00	C_6_H_13_NO_2_	C_9_H_21_NO_2_Si
27	31.691	L-ornithine	132.16	420.25	95.20	C_5_H_12_N_2_O_2_	C_17_H_44_N_2_O_2_Si_4_
28	10.887	L-serine	105.09	249.12	93.85	C_3_H_7_NO_3_	C_9_H_23_NO_3_Si_2_
29	23.045	L-threonic acid	136.10	424.20	93.96	C_4_H_8_O_5_	C_16_H_40_O_5_Si_4_
30	15.997	L-threonine	119.12	335.18	93.52	C_4_H_9_NO_3_	C_13_H_33_NO_3_Si_3_
31	43.819	L-tryptophan	204.22	420.77	90.83	C_11_H_12_N_2_O_2_	C_20_H_36_N_2_O_2_Si_3_
32	43.259	Linoleic acid	280.45	352.28	96.06	C_18_H_32_O_2_	C_21_H_40_O_2_Si
33	20.675	L-5-oxoproline	129.11	273.12	95.39	C_5_H_7_NO_3_	C_11_H_23_NO_3_Si_2_
34	19.916	Malic acid	134.09	350.14	92.41	C_4_H_6_O_5_	C_13_H_30_O_5_Si_3_
35	40.836	Myo-inositol	180.16	612.30	92.69	C_6_H_12_O_6_	C_24_H_60_O_6_Si_6_
36	38.496	Palmitic acid	256.42	328.28	93.58	C_16_H_32_O_2_	C_19_H_40_O_2_Si
37	39.104	Scyllo-inositol	180.16	612.30	93.49	C_6_H_12_O_6_	C_24_H_60_O_6_Si_6_
38	12.886	Succinic acid	118.09	262.11	91.01	C_4_H_6_O_4_	C_10_H_22_O_4_Si_2_
39	44.24	Stearic acid	284.48	356.31	93.51	C_18_H_36_O_2_	C_21_H_44_O_2_Si
40	10.328	Urea	60.06	204.11	92.73	CH_4_N_2_O	C_7_H_20_N_2_OSi_2_

**Table 5:** The content of targeted metabolites in pectoralis major.

Metabolites	Authentic Village Chicken		Broiler (Cobb)		Colored Broiler (Hubbard)		Spent Laying Hen (Dekalb)	
	µg/mg	RSD (%)	µg/mg	RSD (%)	µg/mg	RSD (%)	µg/mg	RSD (%)
D-allose ^2^	57.9 ± 3.1 ^b^	5.3	n/d	-	94.0 ± 8.8 ^a^	9.4	n/d	-
L-phenylalanine ^1^	18.2 ± 2.4 ^b^	13.1	36.8 ± 7.4 ^a^	20.1	16.8 ± 1.8 ^b^	10.9	19.0 ± 2.5 ^b^	13.0
Linoleic acid ^3^	60.1 ± 7.6 ^b^	12.7	108.2 ± 5.1 ^a^	4.7	54.7 ± 7.9 ^b^	14.4	50.6 ± 6.2 ^b^	12.2
L-leucine ^1^	15.6 ± 1.3 ^b^	8.1	n/d	-	n/d	-	37.8 ± 3.2 ^a^	8.6
L-valine ^1^	41.6 ± 5.2 ^a^	12.5	17.5 ± 3.6 ^b^	20.4	39.3 ± 4.7 ^a^	12.0	11.1 ± 1.4 ^b^	12.9
Stearic acid ^3^	33.8 ± 7.6 ^c^	14.1	57.9 ± 6.3 ^b^	10.9	101.1 ± 5.5 ^a^	5.4	20.9 ± 1.5 ^d^	7.3

Significantly different for means within a row with different superscript letters ^a–d^, (*p* < 0.05). ^1^ Calculated results were considered with the % recovery of L-norleucine in muscle (≥105.9). ^2^ Calculated results were considered with the % recovery of ribitol in muscle (≥97.0%). ^3^ Calculated results were considered with the % recovery of heptadecanoic acid in muscle (≥96.0%). n/d indicated for not detected.

**Table 6:** The content of targeted metabolites in chicken serum.

Metabolites	Authentic Village Chicken		Broiler (Cobb)		Colored Broiler (Hubbard)		Spent Laying Hen (Dekalb)	
	µg/mL	RSD (%)	µg/mL	RSD (%)	µg/mL	RSD (%)	µg/mL	RSD (%)
Arachidonic acid ^3^	38.3 ± 1.5 ^b^	3.9	69.2 ± 5.4 ^a^	7.9	41.5 ± 3.7 ^b^	9.0	21.4 ± 1.6 ^c^	3.9
Cysteine ^1^	n/d	-	32.6 ± 2.1	6.5	n/d	-	n/d	-
D-glucose ^2^	2314.6 ± 99.3 ^b^	4.3	3398.5 ± 44.7 ^a^	1.3	1252.2 ± 96.5 ^d^	7.7	1584.8 ± 79.2 ^c^	8.7
Fumaric acid ^2^	12.0 ± 1.0	8.7	n/d	-	n/d	-	n/d	-
Linoleic acid ^3^	26.1 ± 1.9 ^c^	7.2	31.9 ± 1.8 ^bc^	5.7	66.9 ± 6.5 ^a^	9.7	33.2 ± 3.1 ^b^	9.4
L-aspartic acid ^3^	30.4 ± 3.0 ^b^	9.8	24.9 ± 2.0 ^c^	8.0	45.1 ± 3.0 ^a^	6.7	n/d	-
L-leucine ^1^	83.6 ± 6.7 ^a^	8.0	36.9 ± 3.4 ^c^	9.2	53.2 ± 4.1 ^b^	7.6	36.4 ± 2.5 ^c^	6.8
Stearic acid ^3^	37.3 ± 3.1 ^c^	8.3	46.3 ± 4.2 ^b^	9.0	105.9 ± 7.7 ^a^	8.3	50.4 ± 4.0 ^b^	7.8
Urea ^1^	90.6 ± 4.0 ^a^	4.4	43.4 ± 4.1 ^c^	9.5	42.3 ± 3.2 ^c^	7.6	64.8 ± 3.8 ^b^	5.8

Significantly different for means within a row with different superscript letters ^a–d^, (*p* < 0.05). ^1^ Calculated results were considered with the % recovery of L-norleucine in serum (≥92.4%). ^2^ Calculated results were considered with the % recovery of ribitol in serum (≥117.0%). ^3^ Calculated results were considered with the % recovery of heptadecanoic acid in serum (≥106.0). n/d indicated for not detected.

## Data Availability

The data presented in this study are available on request from the corresponding author. The data are not publicly available due to the rules and regulations of University Putra Malaysia (UPM).

## Supplementary Materials

The following are available online at https://www.mdpi.com/article/10.3390/foods10092174/s1, Figure S1: Photos of chicken breeds. Figure S2: Photos of collected pectoralis major and serum. Figure S3: Total ion chromatograms for pectoralis major. Figure S4: Total ion chromatograms for chicken serum. Table S1: Information of chicken samples. Table S2: Performance of GC–MS method. Table S3: List of annotated metabolites for pectoralis major. Table S4: List of annotated metabolites for chicken serum. Table S5: Calibration curve, linear range, limit of detection, and quantification of GC–MS method. Table S6: Repeatability and recovery of GC–MS method. Table S7: Comparison of fold intensity values of metabolites in pectoralis major. Table S8: Comparison of fold intensity values of metabolites in chicken serum. Table S9: Nutritional composition of feeds.
