# Supplementary material for: Muscle and Serum Metabolomics for Different Chicken Breeds under Commercial Conditions by GC–MS
Source: Foods. 2021 Sep 14;10(9):2174. doi: 10.3390/foods10092174 (PMC8467607; doi:10.3390/foods10092174)
Supplement: Supplementary file 1 [file foods-10-02174-s001.zip › foods-1351663-supplementary.pdf]

Supplement document

Figure S1: The photo of four chicken breeds purchased from commercial farms: Malaysian village chicken (a), colored broiler Hubbard (b), broiler Cobb (c), and spent laying chicken Dekalb (d).

Figure S2: The photo of pectoralis major muscle and serum collected in this study.

Figure S3: Total ion chromatogram for pectoralis major samples.

Figure S4: Total ion chromatogram for chicken serum samples.

Table S1: Information of chicken breed samples collected.

Table S2: Method performance on precision and retention time of GC−MS method, RSD (%).

Table S3: List of annotated metabolites from GC−MS analysis for pectoralis major. (n=5)

Table S4: List of annotated metabolites from GC−MS analysis for chicken serum. (n=5)

Table S5: Calibration curves, linear range, limit of detection, and quantification measured by GC−MS method for pectoralis major muscle (μg/mg) and serum (μg/ml).

Table S6. Repeatability and recovery of GC−MS method for pectoralis major muscle (μg/mg) and serum (μg/ml).

Table S7. Comparison of fold intensity values of tentative metabolites in pectoralis major from any two breeds between clusters.

Table S8. Comparison of fold intensity values of tentative metabolites in chicken serum from any two breeds between clusters.

Table S9: Nutrient composition (%) of commercial feeds from the respective commercial farms.

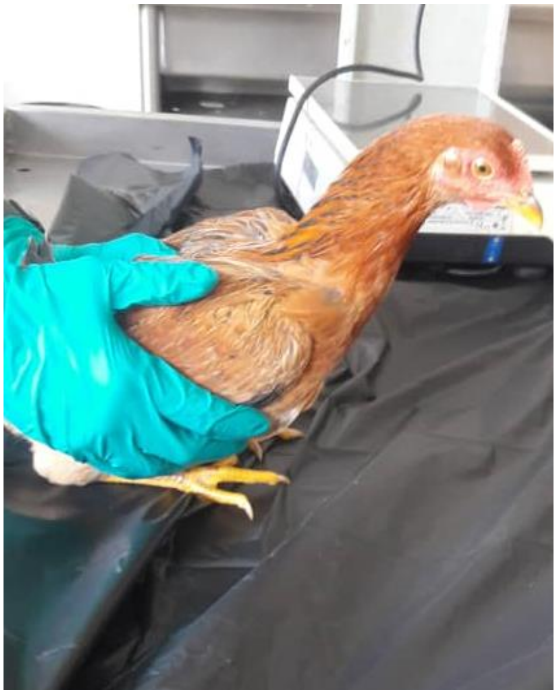

(a)

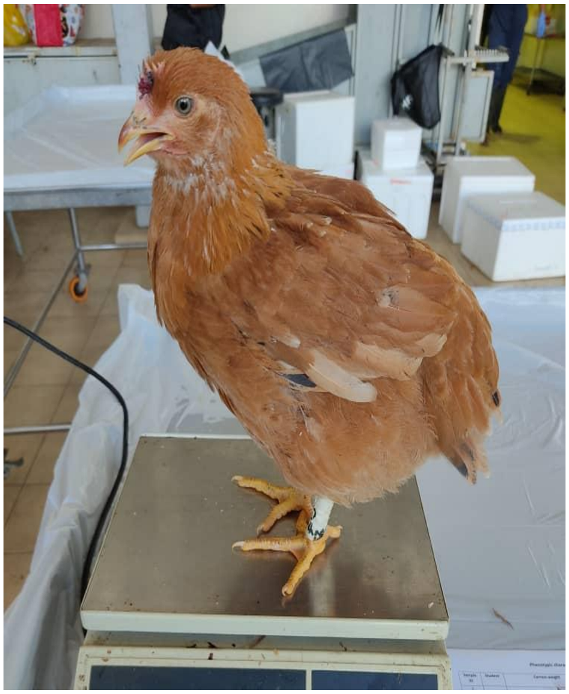

(b)

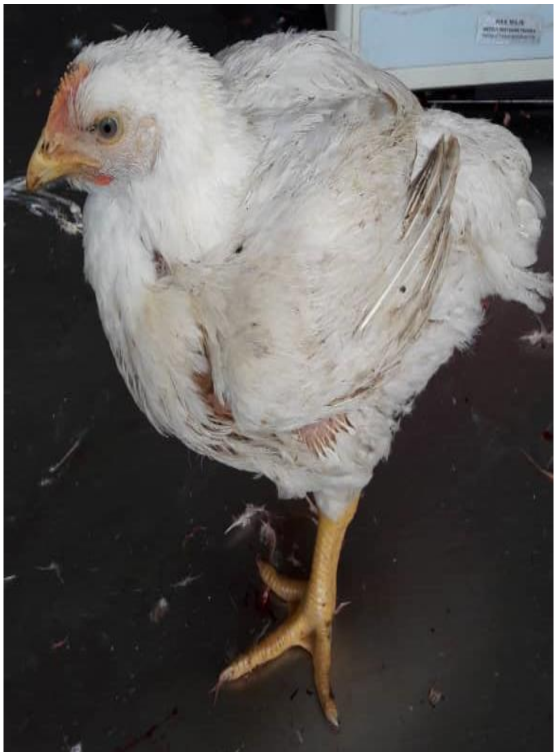

(c)

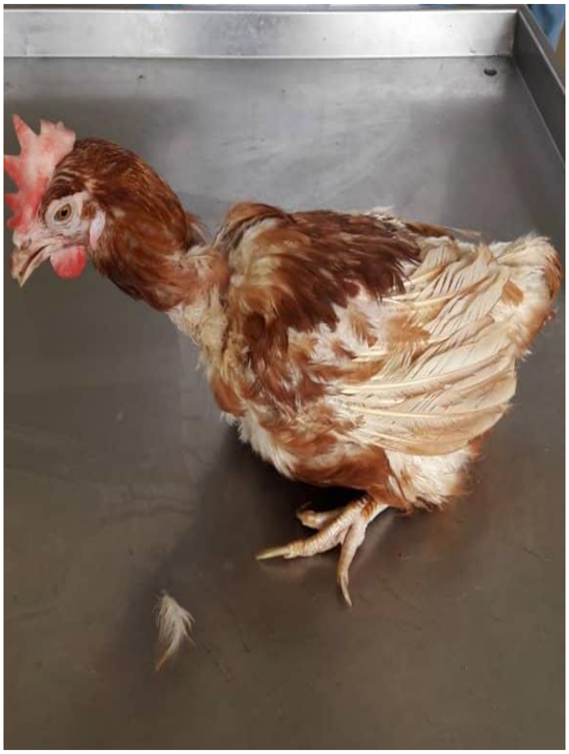

(d)

Figure S1: The photo of four chicken breeds purchased from commercial farms: local village chicken (a), colored broiler–Hubbard (b), broiler–Cobb (c), and spent laying hen–Dekalb (d).

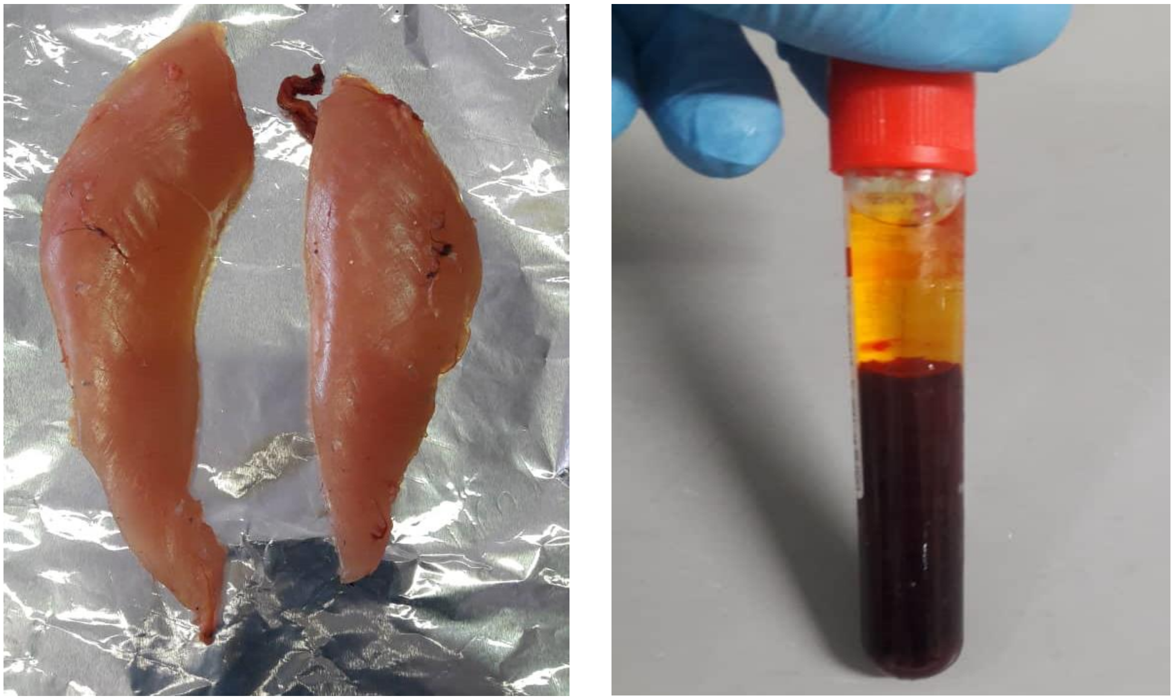

(a) (b)

Figure S2: The photo of pectoralis major muscle (a) and serum (b) collected in this study.

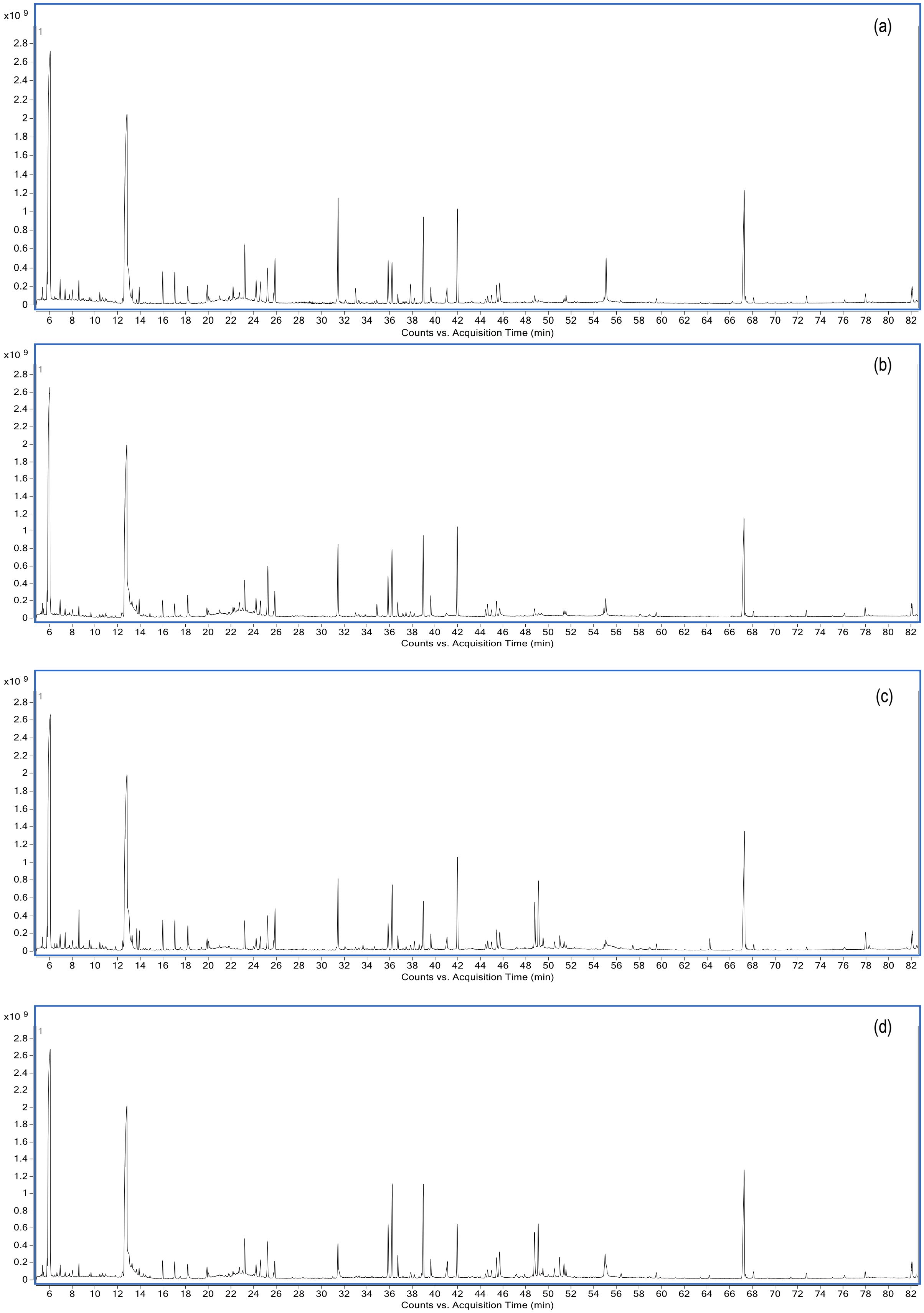

Figure S3. Total ion chromatograms for pectoralis major muscle of different chicken breeds by using GC–MS analysis. (a) village chicken, (b) colored broiler–Hubbard, (c) broiler–Cobb, (d) Spent laying hen–Dekalb

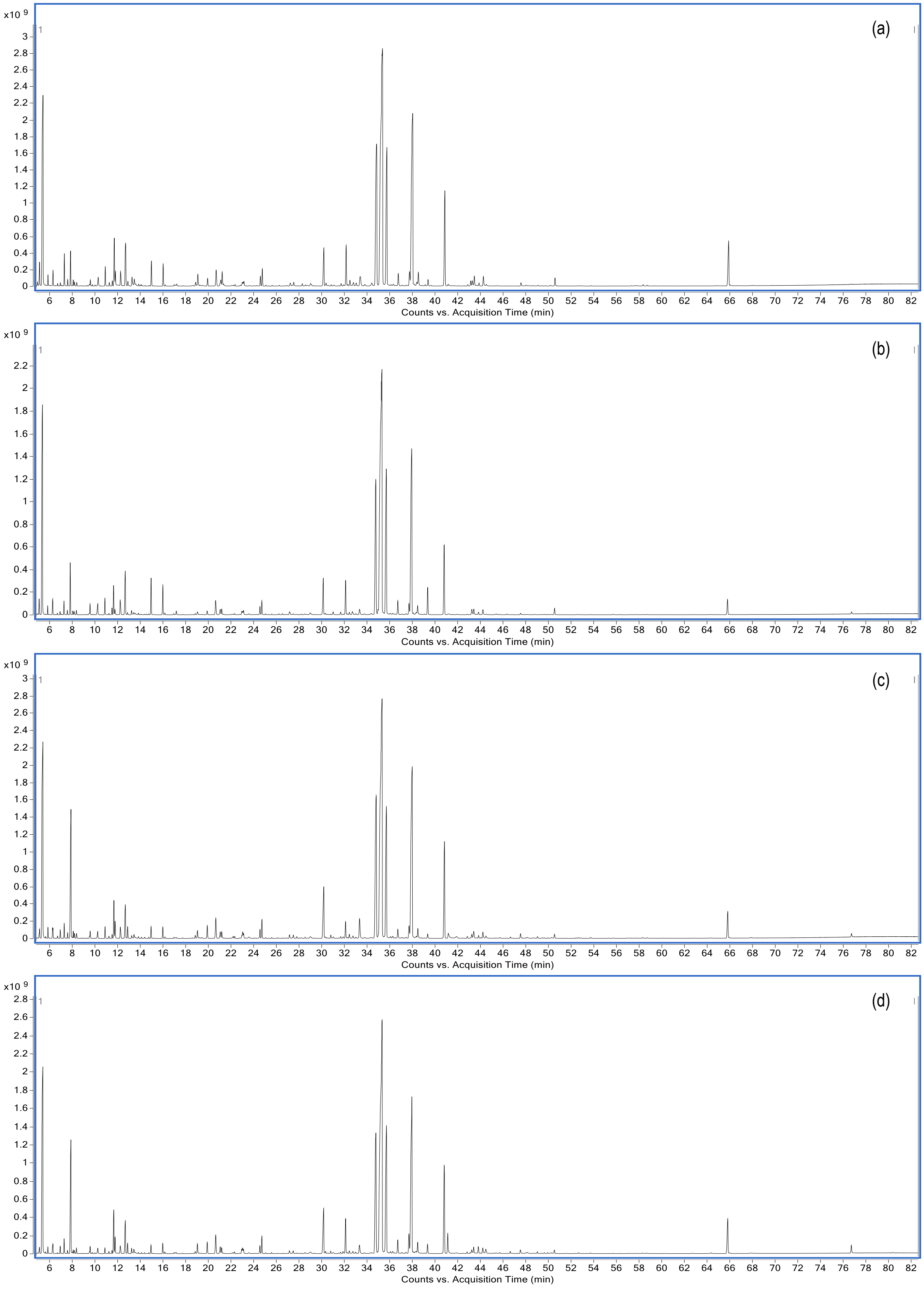

Figure S4. Total ion chromatograms for serum of different chicken breeds by using GC−MS analysis. (a) village chicken, (b) colored broiler−Hubbard, (c) broiler−Cobb, (d) Spent laying hen−Dekalb

Table S1: Information of chicken breed samples collected in this study.

No	Type of chicken	Market age	Market size	Type of feeding	Farm	Rearing condition	Temperature (°C)	Humidity (%)
1	village chicken	14 weeks	1010.5 ± 47.6 g	Commercial feed (Gold coin)	Commercial farm Pahang Tua, Pahang	Open house floor system	25 - 28	50 - 60
2	Colored broiler (Hubbard)	10 weeks	2055.5 ± 66.4 g	Commercial feed (Gold coin)	Commercial farm Permatang Tinggi, Penang	Open house floor system		
3	Broiler (Cobb)	6 weeks	2487.5 ± 53.5 g	Commercial feed (Gold coin)	Commercial farm Bukit Mertajam, Penang	Closed house floor system		
4	Spent laying chicken (Dekalb)	72 weeks	1847.9 ± 58.7 g	Commercial feed (Gold coin layer)	Commercial farm Butterworth, Penang	Closed house cages system		

Table S2. Method Performance on Precision and retention time of GC–MS method, RSD (%)

No	Name of metabolites	Precision (n=5)	RSD of Retention Time
1	3–hydroxybutyric acid	7.17	0.04
2	beta-alanine	6.78	0.05
3	4–hydroxybutanoic acid	8.35	0.02
4	Adenosine	5.35	0.005
5	Ascorbic acid	5.76	0.01
6	Succinic acid	9.10	0.05
7	D–allose	8.12	0.007
8	Glycerol monostearate	7.39	0.007
9	Glycine	7.61	0.03
10	L–aspartic acid	8.84	0.01
11	L–lactic acid	6.53	0.08
12	L–alanine	8.63	0.08
13	L–glutamic acid	7.12	0.01
14	L–leucine	6.21	0.04
15	L–phenylalanine	7.66	0.01
16	L–serine	9.09	0.06
17	L–threonine	6.78	0.05
18	L–valine	8.47	0.07
19	Linoleic acid	6.87	0.01
20	Mannose	8.26	0.01
21	Myo–inositol	9.18	0.006
22	Niacinamine	7.88	0.06
23	Oleic acid	7.12	0.03
24	Palmitic acid	7.67	0.01
25	Panthothenic acid	6.04	0.01
26	Phosphoric acid	8.74	0.03
27	Stearic acid	7.98	0.02
28	Talose	6.47	0.007

Table S3. List of annotated metabolites from GC−MS analysis for pectoralis major. (n=5)

Metabolite	Metabolites group	Authentic village chicken					Broiler (Cobb)					Colored broiler (Hubbard)					Spent laying hen (Dekalb)				
		1	2	3	4	5	1	2	3	4	5	1	2	3	4	5	1	2	3	4	5
1−monooleoylglycerol	Other	nd	nd	nd	nd	nd	0.179	0.184	0.188	0.167	0.161	nd	nd	nd	nd	nd	0.145	0.128	0.141	0.162	0.183
11−octadecenoic acid	Fatty acid	0.269	0.242	0.218	0.238	0.201	0.169	0.182	0.176	0.178	0.127	0.192	0.167	0.170	0.183	0.152	0.237	0.241	0.197	0.223	0.204
2−hydroxybutyric acid	Organic acid	0.117	0.112	0.111	0.121	0.124	0.004	0.002	0.008	0.006	0.003	0.087	0.076	0.073	0.071	0.078	0.054	0.033	0.030	0.051	nd
2−propenoic acid	organic acid	nd	0.010	0.008	0.017	0.009	0.014	0.010	0.007	0.008	0.010	nd	nd	nd	nd	nd	nd	nd	nd	nd	nd
3−hydroxybutyric acid	Organic acid	0.568	0.514	0.493	0.523	0.480	1.982	2.012	1.866	1.931	1.847	1.127	1.146	1.102	1.111	1.076	0.241	0.205	0.175	0.256	0.183
4−hydroxybutanoic acid	Fatty acid	0.169	0.147	0.183	0.171	0.164	0.014	0.018	0.014	0.016	0.012	0.103	0.098	0.084	0.096	0.094	0.027	0.035	0.028	0.019	0.022
Adenosine	Other	nd	0.012	nd	0.009	0.01	0.477	0.495	0.507	0.486	0.441	nd	nd	nd	nd	nd	nd	nd	nd	nd	nd
Arachidonic acid	Fatty acid	0.133	0.142	0.126	0.148	0.138	0.074	0.062	0.078	0.085	0.064	nd	nd	nd	nd	nd	0.085	0.057	0.068	0.052	0.047
Ascorbic acid	Vitamin	nd	nd	nd	nd	nd	0.527	0.573	0.515	0.546	0.531	0.023	0.066	0.024	0.043	0.027	nd	nd	nd	nd	nd
Beta−alanine	Amino acid	0.184	0.137	0.124	0.116	0.117	0.426	0.487	0.541	0.419	0.493	nd	nd	nd	nd	nd	0.581	0.476	0.523	0.488	0.461
Cholest−4−en−3−ol, (3.beta.)	Other	nd	0.007	0.018	0.014	0.006	0.024	0.038	0.034	0.020	0.025	0.007	0.004	0.008	0.008	0.005	nd	nd	nd	nd	nd
Creatinine	Other	0.419	0.452	0.423	0.471	0.404	0.116	0.107	0.118	0.123	0.092	0.146	0.151	0.179	0.148	0.157	0.047	0.041	0.032	0.039	0.042
Cholesterol	Other	2.938	2.842	2.472	2.612	2.513	1.321	1.254	1.471	1.342	1.277	2.270	2.149	2.173	2.027	2.113	2.145	2.087	2.413	2.080	2.174
Cholestan−3−ol, (3.beta.,5.alpha.)	Other	0.064	0.048	0.039	0.072	0.047	0.195	0.146	0.172	0.134	0.156	nd	nd	nd	nd	nd	nd	nd	nd	nd	nd
D−talose	Sugar	0.143	0.168	0.145	0.172	0.169	0.036	0.047	0.028	0.024	0.031	0.114	0.117	nd	0.136	0.129	0.126	0.116	0.132	0.124	0.108
D−allose	Sugar	0.176	0.182	0.167	0.170	0.164	nd	nd	nd	nd	nd	0.389	0.375	0.391	0.382	0.376	nd	nd	nd	nd	nd
D−glucose	Sugar	1.073	1.045	1.184	1.093	1.116	1.082	1.024	0.946	0.983	0.997	0.857	0.891	0.866	0.821	0.846	0.820	0.864	0.914	0.847	0.829
D−mannose	Sugar	0.315	0.370	0.362	0.289	0.272	1.889	1.807	1.791	1.747	1.822	1.163	1.192	1.138	1.102	1.048	0.514	0.488	0.438	0.476	0.502
D−galactose	Sugar	nd	nd	nd	nd	nd	nd	nd	nd	nd	nd	nd	nd	nd	nd	nd	0.027	0.034	0.027	0.018	0.022
D−mannitol	Sugar alcohol	nd	nd	nd	nd	nd	0.014	0.017	0.007	0.009	0.013	nd	nd	nd	nd	nd	nd	nd	nd	nd	nd
D−sorbitol	Sugar alcohol	0.033	0.034	0.030	0.021	0.037	nd	nd	nd	nd	nd	nd	nd	nd	nd	nd	nd	nd	nd	nd	nd
Elaidic acid	Fatty acid	nd	nd	nd	nd	nd	0.007	0.006	0.009	0.008	0.005	nd	nd	nd	nd	nd	0.004	0.002	nd	0.007	0.009
Glycerol monostearate	Other	0.119	0.104	0.098	0.121	0.137	0.219	0.232	0.216	0.244	0.224	0.107	0.079	0.096	0.085	0.073	0.102	0.098	0.121	0.113	0.088
Glycine	Amino acid	0.017	0.025	0.014	0.013	0.008	0.019	0.017	0.013	0.014	0.008	0.024	0.014	0.010	0.027	0.012	0.016	0.025	0.020	0.027	0.024
Inosine	Other	nd	nd	nd	nd	nd	5.586	5.279	5.361	5.472	5.702	nd	nd	nd	nd	nd	nd	nd	nd	nd	nd
Linoleic acid	Fatty acid	0.083	0.092	0.084	0.078	0.086	0.349	0.327	0.361	0.344	0.303	0.074	0.086	0.087	0.073	0.092	0.097	0.095	0.075	0.066	0.075
L−lactic acid	Organic acid	17.318	16.204	16.683	16.064	17.346	3.452	3.433	3.107	3.126	3.436	8.086	8.177	8.208	7.103	7.088	3.791	3.273	2.586	3.441	3.612
L−3−methylhistidine	Amino acid	nd	nd	nd	nd	nd	nd	nd	nd	nd	nd	nd	nd	nd	nd	nd	0.029	0.024	0.017	0.032	0.028

L−alanine	Amino acid	0.036	0.046	0.041	0.034	0.031	0.903	0.847	0.910	0.867	0.809	0.235	0.214	0.233	0.187	0.249	0.180	0.164	0.151	0.253	0.195
L−aspartic acid	Amino acid	nd	nd	nd	nd	nd	0.017	0.015	0.018	0.012	0.014	nd	nd	nd	nd	nd	nd	nd	nd	nd	nd
L−glutamic acid	Amino acid	3.194	3.276	3.205	3.284	3.249	1.249	1.276	1.203	1.084	1.211	1.810	1.876	1.785	1.741	1.726	0.876	0.945	0.912	0.868	0.904
L−leucine	Amino acid	0.064	0.087	0.081	0.068	0.079	nd	nd	nd	nd	nd	nd	nd	nd	nd	nd	0.216	0.257	0.241	0.230	0.209
L−lysine	Amino acid	nd	nd	nd	nd	nd	0.057	0.054	0.041	0.033	0.037	nd	nd	nd	nd	nd	nd	nd	nd	nd	nd
L−phenylalanine	Amino acid	0.089	0.082	0.074	0.078	0.088	0.428	0.464	0.412	0.397	0.381	0.074	0.098	0.076	0.086	0.081	0.102	0.058	0.086	0.071	0.084
L−serine	Amino acid	0.273	0.241	0.267	0.246	0.278	0.042	0.047	0.053	0.058	0.060	0.162	0.197	0.184	0.173	0.192	0.024	0.051	0.037	0.026	0.045
L−threonine	Amino acid	0.782	0.829	0.844	0.796	0.814	0.168	0.154	0.157	0.147	0.173	0.488	0.394	0.362	0.481	0.398	0.145	0.132	0.137	0.149	0.152
L−valine	Amino acid	0.262	0.251	0.254	0.197	0.289	0.087	0.081	0.071	0.084	0.065	0.216	0.202	0.293	0.195	0.207	0.067	0.098	0.083	0.085	0.072
Myo−inositol	Sugar alcohol	1.094	1.147	1.058	1.062	1.193	4.801	4.914	4.830	4.871	4.772	1.423	1.301	1.317	1.439	1.407	1.344	1.285	1.271	1.346	1.308
Niacinamide	Vitamin	0.198	0.240	0.176	0.202	0.237	0.056	0.047	0.058	0.043	0.048	0.162	0.169	0.160	0.157	0.162	0.071	0.067	0.053	0.078	0.065
Oleic acid	Fatty acid	0.242	0.239	0.273	0.246	0.237	0.108	0.147	0.117	0.125	0.136	0.671	0.733	0.809	0.786	0.746	0.756	0.825	0.802	0.793	0.788
Palmitic acid	Fatty acid	0.445	0.406	0.411	0.436	0.516	1.054	1.178	1.084	0.946	1.102	0.261	0.183	0.149	0.185	0.162	0.274	0.346	0.307	0.222	0.408
Pantothenic acid	Vitamin	nd	nd	nd	nd	nd	0.273	0.286	0.284	0.320	0.358	nd	nd	nd	nd	nd	nd	nd	nd	nd	nd
Petroselinic acid	Fatty acid	nd	nd	nd	nd	nd	nd	nd	nd	nd	nd	nd	nd	nd	nd	nd	0.007	0.007	0.010	0.008	0.012
Phosphoric acid	Other	1.258	1.230	1.247	1.181	1.174	0.238	0.282	0.309	0.217	0.194	1.431	1.328	1.272	1.341	1.330	0.308	0.351	0.286	0.245	0.311
Propionic acid	Other	0.178	0.165	0.184	0.173	0.177	0.062	0.057	0.069	0.063	0.052	0.125	0.117	0.148	0.133	0.145	0.012	0.008	0.017	0.010	0.013
Stearic acid	Fatty acid	0.172	0.165	0.141	0.176	0.183	0.291	0.287	0.264	0.272	0.263	0.404	0.410	0.384	0.412	0.475	0.105	0.112	0.108	0.089	0.115
Succinic acid	Organic acid	0.458	0.476	0.428	0.466	0.413	0.946	1.025	0.875	0.884	0.986	nd	nd	nd	nd	nd	0.078	0.064	0.076	0.062	0.051
Urea	Other	nd	nd	nd	nd	nd	nd	nd	nd	nd	nd	0.007	0.005	0.01	0.007	0.003	0.008	nd	0.006	0.003	0.004
Uridine	Other	nd	nd	nd	nd	nd	0.040	0.032	0.028	0.034	0.019	nd	nd	nd	nd	nd	nd	nd	nd	nd	nd

Table S4. List of annotated metabolites from GC−MS analysis for chicken serum. (n=5)

Metabolite	Metabolites group	Authentic village chicken					Broiler (Cobb)					Colored broiler (Hubbard)					Spent laying hen (Dekalb)				
		1	2	3	4	5	1	2	3	4	5	1	2	3	4	5	1	2	3	4	5
1−aminocyclopropane carboxylic acid,	Other	0.013	0.007	0.016	0.008	0.009	nd	nd	nd	nd	nd	nd	nd	nd	nd	nd	nd	nd	nd	nd	nd
2−hydroxybutyric acid	organic acid	0.073	0.081	0.071	0.067	0.062	0.131	0.124	0.108	0.117	0.135	0.414	0.348	0.403	0.427	0.385	0.305	0.296	0.287	0.312	0.309
2,3-butanediol	sugar alcohol	0.016	0.013	0.019	0.007	0.011	nd	nd	nd	nd	nd	nd	nd	nd	nd	nd	nd	nd	nd	nd	nd
2,3−dihydroxybutanoic acid	organic acid	0.016	0.007	0.018	0.013	0.010	0.023	0.017	0.028	0.026	0.014	0.217	0.204	0.271	0.235	0.219	0.172	0.156	0.183	0.174	0.163
3−hydroxybutyric acid	organic acid	2.229	2.104	2.023	2.008	2.143	3.539	3.547	3.504	3.487	3.471	2.447	2.210	2.436	2.408	2.129	1.985	1.867	1.873	1.936	1.908
3,4−dihydroxybutanoic acid	organic acid	0.013	0.025	0.017	0.013	0.026	0.013	0.016	0.014	0.024	0.019	nd	nd	nd	nd	nd	0.010	0.009	0.012	0.008	0.017
9H−purin−6−ol	Other	nd	nd	nd	nd	nd	0.037	0.025	0.031	0.028	0.024	nd	nd	nd	nd	nd	nd	nd	nd	nd	nd
11−octadecenoic acid	Fatty acid	nd	nd	nd	nd	nd	nd	nd	nd	nd	nd	0.016	0.011	0.013	0.015	0.024	nd	nd	nd	nd	nd
Aminomalonic acid	Amino acid	0.257	0.241	0.239	0.248	0.236	0.408	0.412	0.435	0.417	0.384	nd	nd	nd	nd	nd	nd	nd	nd	nd	nd
Arachidonic acid	Fatty acid	0.064	0.078	0.053	0.068	0.071	0.197	0.148	0.166	0.173	0.143	0.056	0.048	0.052	0.046	0.067	0.025	0.020	0.024	0.014	0.016
Asparagine	Amino acid	nd	nd	nd	nd	nd	nd	nd	nd	nd	nd	0.008	0.013	0.009	0.011	0.018	nd	nd	nd	nd	nd
Beta-alanine	Amino acid	0.026	0.037	0.027	0.021	0.018	0.031	0.024	0.038	0.027	0.023	0.014	0.005	0.008	0.006	0.008	0.007	0.017	0.005	0.009	0.008
Cholesterol	Other	0.475	0.462	0.317	0.360	0.418	0.570	0.491	0.487	0.513	0.545	0.617	0.584	0.662	0.603	0.577	0.608	0.579	0.623	0.611	0.547
Citric acid	organic acid	1.023	0.947	0.969	1.108	1.039	0.946	0.802	0.941	0.831	0.874	2.223	2.153	2.198	2.243	2.097	3.085	2.896	3.002	2.995	2.834
Cysteine	Amino acid	nd	nd	nd	nd	nd	0.118	0.105	0.128	0.101	0.106	nd	nd	nd	nd	nd	nd	nd	nd	nd	nd
D−lyxose	Other	0.064	0.057	0.061	0.055	0.048	0.074	0.062	0.058	0.070	0.069	nd	nd	nd	nd	nd	nd	nd	nd	nd	nd
D−allose	sugar	3.985	3.873	3.489	3.621	3.532	3.941	3.575	3.614	3.818	3.716	2.132	2.408	2.089	2.176	2.203	3.075	3.141	3.068	2.906	2.968
D−altrose	sugar	5.724	5.248	5.390	5.471	5.309	2.588	2.403	2.516	2.531	2.705	3.367	3.085	3.174	3.223	3.286	2.846	2.705	2.698	2.883	2.769
D−arabinose	sugar	0.023	0.018	0.014	0.026	0.037	0.014	0.022	0.017	0.019	0.028	nd	nd	nd	nd	nd	0.031	0.027	0.033	0.038	0.043
D−gluconic acid	Other	0.104	0.132	0.127	0.119	0.101	nd	nd	nd	nd	nd	0.005	0.004	0.006	0.004	0.003	nd	nd	nd	nd	nd
D−glucose	sugar	16.074	15.286	16.683	15.330	16.215	21.330	20.473	21.563	20.802	21.607	6.099	6.275	5.210	6.153	5.899	8.431	9.681	9.305	8.608	9.212
D−sorbitol	sugar alcohol	0.018	0.011	0.006	0.008	0.013	0.030	0.041	0.036	0.028	0.034	0.125	0.143	0.133	0.127	0.138	0.227	0.268	0.197	0.241	0.237
D−talose	sugar	4.459	4.258	3.309	4.296	3.388	3.823	3.756	3.847	3.835	3.728	22.846	20.547	21.835	21.086	21.113	4.258	3.679	4.068	4.113	4.009
Ethanolamine	Other	0.134	0.159	0.174	0.163	0.182	0.038	0.026	0.020	0.034	0.031	0.034	0.028	0.018	0.036	0.022	0.014	0.011	0.008	0.012	0.008
Erythritol	sugar alcohol	0.086	0.074	0.064	0.081	0.079	nd	nd	nd	nd	nd	nd	nd	nd	nd	nd	0.033	0.029	0.037	0.031	0.024
Elaidic acid	Fatty acid	0.289	0.274	0.269	0.271	0.286	0.150	0.162	0.143	0.165	0.131	0.396	0.435	0.408	0.387	0.346	0.688	0.647	0.611	0.573	0.609
Fumaric acid	organic acid	0.326	0.317	0.286	0.291	0.277	nd	nd	nd	nd	nd	nd	nd	nd	nd	nd	nd	nd	nd	nd	nd
Glutaric acid	organic acid	nd	nd	nd	nd	nd	0.104	0.135	0.122	0.107	0.131	nd	nd	nd	nd	nd	nd	nd	nd	nd	nd
Glyceric acid	Other	0.229	0.251	0.276	0.239	0.244	nd	nd	nd	nd	nd	0.030	0.035	0.027	0.038	0.031	0.177	0.136	0.154	0.148	0.169

Glycerol	sugar alcohol	0.222	0.210	0.185	0.192	0.184	0.348	0.352	0.368	0.343	0.317	0.142	0.127	0.121	0.113	0.139	0.132	0.144	0.128	0.100	0.116
Glycine	Amino acid	0.153	0.141	0.139	0.167	0.136	0.014	0.018	0.008	0.016	0.011	0.024	0.016	0.028	0.025	0.013	0.036	0.047	0.025	nd	0.034
Glycolic acid	organic acid	0.004	0.008	0.007	0.003	0.004	0.027	0.016	0.023	0.017	0.022	nd	nd	nd	nd	nd	0.022	0.013	0.016	0.027	0.021
Linoleic acid	Fatty acid	0.186	0.187	0.194	0.202	0.187	0.214	0.207	0.184	0.202	0.197	0.281	0.274	0.269	0.271	0.288	0.215	0.223	0.217	0.204	0.216
L−arabitol	sugar alcohol	nd	nd	nd	nd	nd	0.028	0.031	0.024	0.027	0.019	0.232	0.284	0.206	0.311	0.263	0.113	0.140	0.126	0.142	0.138
L−lactic acid	organic acid	8.152	8.038	7.836	7.904	7.887	2.338	2.409	2.262	2.411	2.682	2.162	1.961	2.086	2.446	2.701	1.845	2.483	1.665	2.160	2.917
L−5−oxoproline	Amino acid	nd	nd	nd	nd	nd	0.640	0.585	0.536	0.612	0.632	nd	nd	nd	nd	nd	0.836	0.825	0.903	0.915	0.852
L−alanine	Amino acid	0.164	0.193	0.174	0.188	0.167	0.053	0.068	0.051	0.064	0.057	0.141	0.106	0.055	0.086	0.129	0.127	0.083	0.061	0.104	0.112
L−aspartic acid	Amino acid	0.217	0.303	0.295	0.224	0.308	0.172	0.146	0.163	0.188	0.179	0.377	0.418	0.541	0.445	0.404	nd	nd	nd	nd	nd
L−glutamic acid	Amino acid	0.395	0.387	0.474	0.489	0.370	0.087	0.065	0.048	0.052	0.067	0.078	0.061	0.058	0.071	0.055	0.008	0.005	0.005	0.007	0.015
L−glutamine	Amino acid	0.806	0.736	0.814	0.744	0.758	1.194	1.208	1.186	1.241	1.203	0.623	0.527	0.511	0.639	0.514	0.308	0.346	0.381	0.368	0.280
L−hydroxyproline	Amino acid	0.245	0.214	0.237	0.261	0.243	0.059	0.056	0.041	0.053	0.062	nd	nd	nd	nd	nd	nd	nd	nd	nd	nd
L−leucine	Amino acid	0.379	0.322	0.291	0.314	0.306	0.009	0.011	0.007	0.016	0.008	0.113	0.104	0.087	0.133	0.081	0.007	0.018	0.009	0.014	0.012
L−lysine	Amino acid	nd	nd	nd	nd	nd	0.008	0.005	0.006	0.009	0.007	nd	nd	nd	nd	nd	nd	nd	nd	nd	nd
L−ornithine	Amino acid	nd	nd	nd	nd	nd	0.032	0.027	0.024	0.026	0.033	0.220	0.241	0.206	0.181	0.232	0.409	0.382	0.344	0.321	0.336
L−phenylalanine	Amino acid	0.184	0.171	0.168	0.173	0.202	0.135	0.151	0.140	0.139	0.143	0.125	0.162	0.146	0.120	0.108	0.176	0.168	0.126	0.185	0.163
L−serine	Amino acid	0.339	0.324	0.354	0.316	0.349	0.120	0.127	0.138	0.142	0.133	0.282	0.248	0.287	0.238	0.194	0.143	0.085	0.120	0.131	0.106
L−threitol	sugar alcohol	nd	nd	nd	nd	nd	0.003	0.006	0.005	0.005	0.007	nd	nd	nd	nd	nd	nd	nd	nd	nd	nd
L−threonic acid	Other	0.055	0.047	0.052	0.041	0.059	0.042	0.038	0.049	0.043	0.045	0.412	0.385	0.426	0.297	0.322	0.132	0.124	0.135	0.164	0.118
L−threonine	Amino acid	0.500	0.485	0.494	0.514	0.523	0.280	0.269	0.276	0.244	0.308	0.253	0.280	0.241	0.268	0.242	0.105	0.122	0.114	0.129	0.130
L−tryptophan	Amino acid	0.039	0.024	0.028	0.021	0.035	0.100	0.108	0.113	0.128	0.104	0.021	0.023	nd	0.036	0.024	nd	nd	nd	nd	nd
L−valine	Amino acid	0.147	0.163	0.152	0.149	0.150	0.147	0.133	0.160	0.138	0.152	0.086	0.051	0.084	0.069	0.053	0.093	0.128	0.106	0.113	0.064
Malic acid	organic acid	0.182	0.174	0.162	0.185	0.179	0.298	0.305	0.314	0.322	0.319	nd	nd	nd	nd	nd	0.154	0.173	0.205	0.167	0.180
Myo−inositol	sugar alcohol	2.869	2.761	2.805	2.740	2.805	4.249	4.108	4.099	4.325	4.213	2.304	2.226	2.141	2.307	2.288	1.874	1.659	1.702	1.883	1.752
Oleic acid	Fatty acid	nd	nd	nd	nd	nd	nd	nd	nd	nd	nd	0.107	0.128	0.076	0.095	0.081	0.093	0.078	0.104	0.086	0.091
Palmitic acid	Fatty acid	0.481	0.450	0.473	0.466	0.492	0.691	0.718	0.723	0.701	0.688	0.135	0.178	0.154	0.166	0.087	0.223	0.249	0.217	0.256	0.242
Pyroglutamic acid	Amino acid	nd	nd	nd	nd	nd	0.005	0.006	0.007	0.005	0.005	nd	nd	nd	nd	nd	nd	nd	nd	nd	nd
Scyllo−inositol	sugar alcohol	0.215	0.207	0.243	0.201	0.218	nd	nd	nd	nd	nd	nd	nd	nd	nd	nd	0.105	0.077	0.081	0.116	0.096
Succinic acid	organic acid	0.103	0.097	0.114	0.087	0.101	0.207	0.194	0.214	0.216	0.223	0.035	0.038	0.031	0.027	0.033	0.014	0.017	0.012	0.009	0.011
Stearic acid	Fatty acid	0.204	0.219	0.207	0.228	0.230	0.342	0.373	0.355	0.348	0.362	0.585	0.573	0.529	0.541	0.486	0.310	0.361	0.283	0.334	0.348
Urea	Other	0.605	0.574	0.586	0.511	0.478	0.123	0.104	0.116	0.107	0.120	0.128	0.110	0.096	0.108	0.087	0.171	0.183	0.174	0.188	0.174
Uridine	Other	0.048	0.035	0.041	0.047	0.038	0.025	0.016	0.024	0.021	0.026	nd	nd	nd	nd	nd	nd	nd	nd	nd	nd
Xylitol	sugar alcohol	nd	nd	nd	nd	nd	0.014	0.016	0.013	0.009	0.018	nd	nd	nd	nd	nd	nd	nd	nd	nd	nd

Table S5. Calibration curves, linear range, limit of detection, and quantification measured by GC−MS method for pectoralis major muscle (μg/mg) and serum (μg/ml).

**Metabolites**	**Standard Curve**	**Correlation Coefficient, R^2**	**Linear Range**	**LOD**	**LOQ**	**Matrix**
Arachidonic acid	y = 4,049,955x - 40,459,873	0.9990	10-1000	2.8	10	Serum
Cysteine	y = 13,938,821x - 136,973,733	0.9989	20-1000	5.8	20	Serum
D-allose	y = 9,539,946x - 100,247,960	0.9992	20-2000	5.5	20	Muscle
D−glucose	y = 12,977,758x - 56,476,414	0.9976	500-8000	138.4	500	Serum
Fumaric acid	y = 13,935,248x - 32,264,388	0.9994	5-500	1.5	5	Serum
L−phenylalaine	y = 18,068,178x - 140,579,623	0.9922	8-500	2.1	8	Muscle
Linoleic acid	y = 5,191,535x - 109,289,208	0.9960	25-500	7.5	25	Muscle
Linoleic acid	y = 7,447,217x - 24,540,154	0.9973	10-500	3	10	Serum
L−aspartic acid	y = 2,457,783x - 7,825,001	0.9998	10-1000	3	10	Serum
L−leucine	y = 6,544,965x - 10,632,712	0.9994	10-400	2.6	10	Muscle
L−leucine	y = 3,631,229x - 2,272,382	0.9985	25-1000	4.1	25	Serum
L−valine	y = 8,898,035x - 20,622,339	0.9999	10-500	1.9	10	Muscle
Stearic acid	y = 9,695,804x - 59,487,465	0.9998	12-500	3.3	12	Muscle
Stearic acid	y = 4,203,419x + 38,389,044	0.9985	15-1000	3.4	15	Serum
Urea	y = 11,284,884x - 124,578,683	0.9987	5-1000	1.5	5	Serum

Table S6. Repeatability and recovery of GC−MS method for pectoralis major muscle (μg/mg) and serum (μg/ml).

Metabolites	Added	Found	Recovery (%)	Repeatability, RSDr (%)	Matrix
Arachidonic acid	200	197.80	98.90	0.99	Serum
	400	367.80	91.95	1.04	
	600	659.80	109.97	0.88	
Cysteine	150	156.30	104.20	1.12	Serum
	300	303.00	100.98	0.73	
	500	498.20	99.64	0.36	
D−allose	150	152.50	101.68	1.24	Muscle
	500	503.30	100.67	0.76	
	1000	1021.40	101.76	0.89	
D−glucose	1000	1054.80	105.48	0.99	Serum
	2000	1989.40	99.11	0.83	
	4000	3743.40	93.59	0.73	
Fumaric acid	50	47.50	95.05	1.06	Serum
	100	101.80	101.83	1.38	
	150	152.00	101.36	1.23	
L−phenylalanine	150	139.50	97.88	1.30	Muscle
	600	654.30	101.74	0.91	
	1000	1073.80	107.98	0.86	
Linoleic acid	100	93.60	93.59	0.73	Muscle
	200	198.50	99.24	0.97	
	300	278.00	92.67	0.86	
Linoleic acid	50	54.40	108.81	1.49	Serum
	100	98.70	98.70	1.25	
	300	290.80	96.92	0.79	
L−aspartic acid	100	98.50	99.00	1.29	Serum
	200	193.50	96.76	1.27	
	400	402.60	100.65	1.13	
L−leucine	50	55.50	110.91	1.21	Muscle
	100	91.20	91.24	1.32	
	380	418.50	110.14	1.03	
L−leucine	100	106.60	106.65	1.19	Serum
	200	187.90	93.90	1.23	
	500	513.50	102.69	0.96	
L−valine	80	81.80	102.23	1.11	Muscle
	160	159.10	99.43	1.06	
	250	253.60	101.46	1.19	
Stearic acid	90	94.50	105.04	1.34	Muscle
	180	177.60	98.68	1.11	
	350	336.80	96.22	0.98	
Stearic acid	90	88.40	98.18	1.27	Serum
	150	154.70	103.13	1.07	
	400	401.40	100.35	0.87	
Urea	200	213.00	106.50	1.20	Serum
	400	427.50	106.85	1.05	
	800	785.00	98.12	1.03	

Table S7. Comparison of fold intensity values of tentative metabolites in pectoralis major from any two breeds between clusters.

Metabolite	VC vs BC		VC vs LD		BH vs BC		BH vs LD		BC vs LD	
	FI	P	FI	P	FI	P	FI	P	FI	P
2−hydroxybutyric acid	25.435	<0.05	2.786	<0.05	16.739	<0.05	1.833	0.05	0.110	<0.05
3−hydroxybutyric acid	0.267	<0.05	2.432	<0.05	0.577	<0.05	5.247	<0.05	9.092	<0.05
4−hydroxybutanoic acid	11.270	<0.05	6.366	<0.05	6.419	<0.05	3.626	<0.05	0.565	0.005
Adenosine	0.021	<0.05	-	-	-	-	-	-	-	-
Ascorbic acid	-	-	-	-	0.068	<0.05	-	-	-	-
Beta−alanine	0.287	<0.05	0.664	<0.05	-	-	-	-	0.619	0.327
Creatinine	3.901	<0.05	10.791	<0.05	1.405	0.05	3.886	<0.05	2.766	<0.05
D−talose	4.801	<0.05	1.315	0.001	2.988	<0.05	0.818	0.682	0.274	<0.05
D−mannose	0.178	<0.05	0.665	<0.05	0.623	<0.05	2.334	<0.05	3.745	<0.05
Glycerol monostearate	0.510	<0.05	1.109	0.244	0.388	<0.05	0.843	0.091	2.174	<0.05
L−lactic acid	5.051	<0.05	5.006	<0.05	2.336	0.05	2.315	<0.05	0.991	0.899
L−alanine	0.043	<0.05	0.199	<0.05	0.258	<0.05	1.186	0.129	4.598	<0.05
L−glutamic acid	2.691	<0.05	3.598	<0.05	1.484	<0.05	1.984	<0.05	1.337	<0.05
L−leucine	-	-	0.329	<0.05	-	-	-	-	-	-
L−phenylalanine	0.197	<0.05	1.025	0.808	0.199	<0.05	1.035	0.752	5.192	<0.05
L−serine	5.019	<0.05	7.131	<0.05	3.492	<0.05	4.962	<0.05	1.421	0.038
L−threonine	5.088	<0.05	5.685	<0.05	2.657	0.05	2.969	<0.05	1.117	0.027
L−valine	3.229	<0.05	3.094	<0.05	2.869	<0.05	2.748	<0.05	0.958	0.631
Linoleic acid	0.251	<0.05	1.037	0.658	0.245	<0.05	1.010	0.914	4.127	<0.05
Myo−inositol	0.230	<0.05	0.847	0.05	0.285	<0.05	1.051	0.085	3.691	<0.05
Niacinamide	4.179	<0.05	3.153	<0.05	3.214	<0.05	2.425	<0.05	0.754	0.011
Oleic acid	1.954	<0.05	0.312	<0.05	5.916	<0.05	0.945	0.134	0.160	<0.05
Palmitic acid	0.413	<0.05	1.422	0.008	0.175	<0.05	0.604	0.01	3.445	<0.05
Phosphoric acid	4.911	<0.05	4.057	<0.05	5.405	<0.05	4.465	<0.05	0.826	0.092
Propionic acid	2.894	<0.05	14.617	<0.05	2.205	<0.05	11.133	<0.05	5.050	<0.05
Stearic acid	0.608	<0.05	1.582	<0.05	1.514	<0.05	3.941	<0.05	2.603	<0.05
Succinic acid	0.475	<0.05	6.770	<0.05	-	-	-	-	14.248	<0.05

Statistically significant was indicated by $P < 0.05$.

Table S8. Comparison of fold intensity values of tentative metabolites in chicken serum from any two breeds between clusters.

Metabolite	VC vs BC		VC vs LD		VC vs BH		BC vs BH		BC vs LD	
	FI	P	FI	P	FI	P	FI	P	FI	P
2−hydroxybutyric acid	0.576	<0.05	0.235	<0.05	0.179	<0.05	0.311	<0.05	0.408	<0.05
2,3−dihydroxybutanoic acid	0.593	0.029	0.075	<0.05	0.056	<0.05	0.094	<0.05	0.127	<0.05
3−hydroxybutyric acid	0.599	<0.05	1.098	0.007	0.903	0.027	1.509	<0.05	1.834	<0.05
Aminomalonic acid	0.594	<0.05	-	-	-	-	-	-	-	-
Arachidonic acid	0.404	<0.05	3.374	<0.05	1.275	0.052	3.156	<0.05	8.354	<0.05
Citric acid	1.157	0.011	0.343	<0.05	0.466	<0.05	0.403	<0.05	0.297	<0.05
D−altrose	2.130	<0.05	1.953	<0.05	1.682	<0.05	0.790	<0.05	0.917	0.007
D−gluconic acid	-	-	-	-	26.500	<0.05	-	-	-	-
D−glucose	0.752	<0.05	1.759	<0.05	2.686	<0.05	3.569	<0.05	2.338	<0.05
D−sorbitol	0.331	<0.05	0.048	<0.05	0.084	<0.05	0.254	<0.05	0.144	<0.05
D−talose	1.038	0.589	0.979	0.764	0.183	<0.05	0.177	<0.05	0.943	0.082
Elaidic acid	1.850	<0.05	0.444	<0.05	0.704	0.002	0.381	<0.05	0.240	<0.05
Ethanolamine	5.450	<0.05	15.321	<0.05	5.884	<0.05	1.080	0.650	2.811	0.002
Glyceric acid	-	-	1.580	<0.05	7.696	<0.05	-	-	-	-
Glycerol	0.575	<0.05	1.602	<0.05	1.547	<0.05	2.692	<0.05	2.787	<0.05
Glycine	10.985	<0.05	4.146	<0.05	6.943	<0.05	0.632	0.059	0.377	0.020
L−arabitol	-	-	-	-	-	-	0.100	<0.05	0.196	<0.05
L−aspartic acid	1.588	0.005	-	-	0.616	0.002	0.388	0.05	-	-
L−glutamic acid	6.630	<0.05	52.875	<0.05	6.548	<0.05	0.988	0.924	7.975	0.05
L−glutamine	0.640	<0.05	2.292	<0.05	1.371	0.05	2.144	<0.05	3.584	<0.05
L−hydroxyproline	4.428	<0.05	-	-	-	-	-	-	-	-
L−lactic acid	3.290	<0.05	3.597	<0.05	3.506	<0.05	1.066	0.924	1.093	0.05
L−leucine	31.608	<0.05	26.867	<0.05	3.112	<0.05	0.098	0.05	0.850	0.495
L−ornithine	-	-	-	-	-	-	0.131	<0.05	0.079	<0.05
L−serine	2.548	<0.05	2.875	<0.05	1.347	0.005	0.528	0.002	1.128	0.223
L−threonic acid	1.171	0.087	0.377	<0.05	0.138	<0.05	0.118	<0.05	0.322	<0.05
L−threonine	1.827	<0.05	4.193	<0.05	1.960	<0.05	1.072	0.188	2.295	<0.05
L−tryptophan	0.266	<0.05	-	-	1.131	0.503	4.254	<0.05	-	-
Linoleic acid	0.952	0.155	0.889	0.05	0.691	<0.05	0.726	<0.05	0.934	0.053
L−5−oxoproline	-	-	-	-	-	-	-	-	0.694	<0.05
Malic acid	0.566	<0.05	1.003	0.951	-	-	-	-	1.772	<0.05
Myo−inositol	0.666	<0.05	1.576	<0.05	1.241	<0.05	1.863	<0.05	2.367	<0.05
Palmitic acid	0.671	<0.05	1.990	<0.05	3.281	<0.05	4.890	<0.05	2.966	<0.05
Scyllo−inositol	-	-	2.282	<0.05	-	-	-	-	-	-
Stearic acid	0.611	<0.05	0.665	0.05	0.401	<0.05	0.656	0.05	1.088	0.112
Succinic acid	0.476	<0.05	7.968	<0.05	3.061	<0.05	6.427	<0.05	16.730	<0.05
Urea	4.832	<0.05	3.094	<0.05	5.206	<0.05	1.078	0.337	0.640	<0.05

Statistically significant was indicated by $P < 0.05$.

Table S9. Nutrient composition (%) of commercial feed from the respective commercial farms.

Nutrient Composition	Malaysian Village Chicken	Colour Broiler (Hubbard)	Broiler (COBB)	Spent Laying Hen (Dekalb)
Crude protein (%)	19.27^{a}	19.63^{a}	19.37^{a}	16.53^{b}
Crude fat (%)	7.45^{a}	7.38^{a}	7.47^{a}	3.58^{b}
Crude Fiber (%)	3.25^{b}	3.14^{b}	3.32^{b}	3.84^{a}
Ash (%)	5.71	5.64	5.68	5.82
Moisture (%)	10.43^{b}	10.51^{b}	10.63^{b}	11.46^{a}
Dry matter (%)	89.57^{a}	89.49^{a}	89.37^{a}	88.54^{b}

Significant different for means within a row with different superscript letters a-b, ($P < 0.05$).
